# Rewiring the Epigenetic Networks in *MLL*-Rearranged Leukemias: Epigenetic Dysregulation and Pharmacological Interventions

**DOI:** 10.3389/fcell.2019.00081

**Published:** 2019-05-15

**Authors:** Anthony K. N. Chan, Chun-Wei Chen

**Affiliations:** Department of Systems Biology, Beckman Research Institute of City of Hope, Duarte, CA, United States

**Keywords:** *MLL*-rearranged, leukemias, epigenetics, MLL, chromatin

## Abstract

Leukemias driven by chromosomal translocation of the mixed-lineage leukemia gene (*MLL* or *KMT2A*) are highly prevalent in pediatric oncology. The poor survival rate and lack of an effective targeted therapy for patients with *MLL*-rearranged (*MLL*-r) leukemias emphasize an urgent need for improved knowledge and novel therapeutic approaches for these malignancies. The resulting chimeric products of *MLL* gene rearrangements, i.e., MLL-fusion proteins (MLL-FPs), are capable of transforming hematopoietic stem/progenitor cells (HSPCs) into leukemic blasts. The ability of MLL-FPs to reprogram HSPCs toward leukemia requires the involvement of multiple chromatin effectors, including the histone 3 lysine 79 methyltransferase DOT1L, the chromatin epigenetic reader BRD4, and the super elongation complex. These epigenetic regulators constitute a complicated network that dictates maintenance of the leukemia program, and therefore represent an important cluster of therapeutic opportunities. In this review, we will discuss the role of MLL and its fusion partners in normal HSPCs and hematopoiesis, including the links between chromatin effectors, epigenetic landscapes, and leukemia development, and summarize current approaches to therapeutic targeting of *MLL*-r leukemias.

## Introduction

In the 1980s, leukemia cases were observed that showed complete phenotypic lineage switching, i.e., patients initially diagnosed as acute lymphocytic leukemia (ALL) relapsed as acute myeloid leukemia (AML) during chemotherapy (Stass et al., 1984; Mirro et al., 1985; Gagnon et al., 1989). Accordingly, the term mixed-lineage leukemia was coined (Mirro et al., 1985; Slany, 2009). Mixed-linage leukemia was reported to be associated with translocation on the long arm (q) of chromosome 11 band q23 (11q23) (Hayashi et al., 1990). Recurring 11q23 translocations are also found in both ALL (Inaba et al., 2013; Roberts and Mullighan, 2015) and AML (Döhner et al., 2015; Papaemmanuil et al., 2016). Guided by these observations, researchers located and cloned an important gene that resides on 11q23 and drives leukemogenesis when it is translocated and fused with other gene partners (Ziemin-van der Poel et al., 1991; Djabali et al., 1992; Gu et al., 1992; Tkachuk et al., 1992). In the initial discoveries, the gene was given different names: *MLL* (myeloid/lymphoid, or mixed-lineage leukemia) (Ziemin-van der Poel et al., 1991), *ALL-1* (Gu et al., 1992), *HRX* (human trithorax, the human homolog of the *Drosophilia trithorax*, *trx*) (Tkachuk et al., 1992), and *trithorax-like* gene (Djabali et al., 1992). Subsequently, it was also re-named as *KMT2A* (lysine methyltransferase 2A), based on its lysine (Lys, K) methyltransferase enzymatic activity. For consistent description of the gene, we will use *MLL* throughout this review article.

Accordingly, leukemias that involve chromosomal rearrangement of *MLL* are called *MLL*-rearranged (*MLL*-r) leukemias (Rowley, 1993; Arber et al., 2016; Krivtsov et al., 2017). In adults, *MLL*-r leukemia accounts for approximately 5% of ALL cases (Marchesi et al., 2011) and 5–10% of AML cases (Krivtsov and Armstrong, 2007; Chowdhury and Brady, 2008; Chen and Armstrong, 2015). *MLL*-r leukemias are more prevalent in infant patients (<1 year of age), in which approximately 70% of infants with ALL are diagnosed as having 11q23 rearrangements (Chen et al., 1993; Heerema et al., 1999; Muntean and Hess, 2012). AML is less common than ALL in infants, in which about 50–66% of infants with AML have 11q23 translocations (Sorensen et al., 1994; Martinez-Climent et al., 1995; Hilden et al., 1997; Satake et al., 1999). Prognosis is poor for infants with *MLL*-r ALL, who have worse outcomes than do older children (>12 months) (Pieters et al., 2007). The five-year event-free survival rate of non-*MLL*-r ALL can approach 60–96% in infants (<1 year of age), but is significantly lower in *MLL*-r infant ALL patients—only 34–39% depending on treatment protocols (Hilden et al., 2006; Tomizawa et al., 2007). In addition, approximately 2–15% of cancer patients receiving chemotherapeutic drugs that target DNA-topoisomerase II develop treatment-related AML with 11q23 translocations (Super et al., 1993; Broeker et al., 1996; Greaves, 1997; Felix, 1998). These situations emphasize the unmet need for a deeper understanding of the underlying biology and novel therapeutic approaches for *MLL*-r leukemia.

Surprisingly, comprehensive genomic studies revealed that the genomes of patient-derived *MLL*-r leukemia cells displayed remarkable stability with only a few genetic alterations (Mullighan et al., 2007; Radtke et al., 2009). This suggests that *MLL*-r leukemias are largely driven by epigenetic dysregulation (Radtke et al., 2009; Bernt and Armstrong, 2011). Epigenetics is the study of heritable and reversible control of gene expression that is not dependent on the DNA sequence. In eukaryotic nuclei, DNA molecules are wrapped around histone cores consisting of eight histone proteins—H2A, H2B, H3, and H4 (two copies of each protein are present in the core). Approximately 146 bp of DNA surrounds each octameric histone core to form a nucleosome, the basic structural unit of chromatin. The nucleosomal units are packed and condensed further to form chromatin. The N-terminal peptide tails of H3 and H4 protrude from the histone core, and can be post-translationally modified in various ways (e.g., acetylation, methylation, phosphorylation, ubiquitination, sumoylation) inside eukaryotic cells (Bannister and Kouzarides, 2011). These modifications can be added by epigenetic writers, interpreted by epigenetic readers, and removed by epigenetic erasers (Arrowsmith et al., 2012; Musselman et al., 2012; Seto and Yoshida, 2014; Zhang et al., 2015). Specific modifications or epigenetic histone marks have differential effects on gene expression. For example, acetylated histone marks (e.g., H3K9ac and H3K27ac) are usually associated with gene activation (Krejčí et al., 2009; Creyghton et al., 2010; Hawkins et al., 2011; Hezroni et al., 2011). In contrast, methylated modifications are context-dependent: for instance, methylation on H3K4 or H3K79 is associated with gene activation (Schübeler et al., 2004), whereas methylation on H3K9 or H3K27 is associated with gene silencing (Musselman et al., 2012). In this review, we will introduce the mechanistic roles of MLL in normal hematopoiesis and *MLL*-r leukemia, describe current therapeutic targets in *MLL*-r leukemia, with an emphasis on chromatin epigenetic regulators, and discuss the potential of using combined epigenetic targeting strategies to treat *MLL*-r leukemia.

## MLL in Normal Hematopoiesis and **MLL**-r Leukemias

### MLL Protein Structure and Function

**FIGURE 1:**
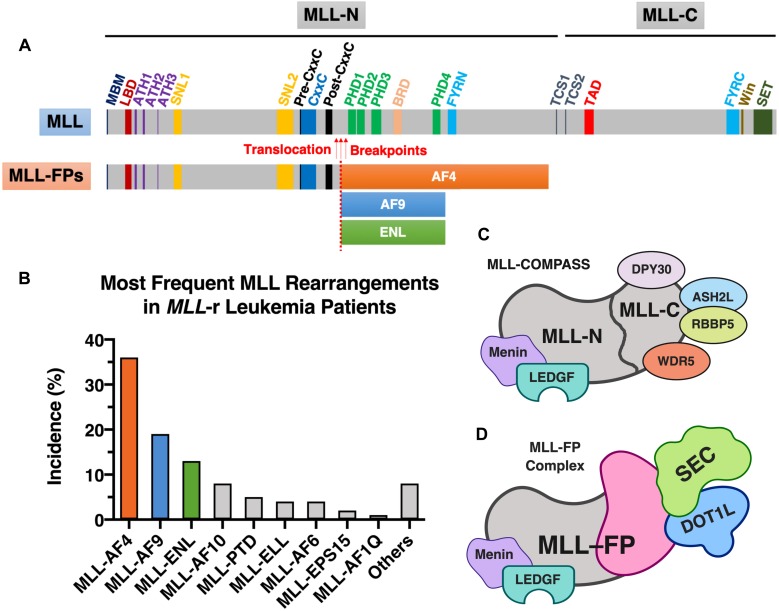
MLL and MLL-FPs. **(A)** Schematic of domain architecture of wild-type MLL and MLL-FPs. The wild-type canonical form of human MLL protein (UniProt ID: Q03164) has a total of 3,969 amino acids in length and contains several functional domains and important sites (drawn to scale): high-affinity Menin-binding motif (MBM, residue 6–10) (Yokoyama et al., 2005), LEDGF-binding domain (LBD, residue 109–153) (Yokoyama and Cleary, 2008), AT-Hook1/2/3 (ATH1, residue 169–180; ATH2, residue 217–227; ATH3, residue 301–309; UniProt annotations of Q03164); nuclear-localization signal 1/2 (SNL1, residue 400–443; SNL2, 1008–1106) (Ayton et al., 2004), pre-CxxC region (residue 1149–1154) (Muntean et al., 2010), CxxC domain (residue 1147–1242) (Bach et al., 2008), post-CxxC (residue 1298–1337) (Muntean et al., 2010), plant homology domain 1/2/3/4 (PHD1, residue 1431–1482; PHD2, residue 1479–1533, PHD3, residue 1566–1627; PHD4, residue 1931–1978; UniProt annotations of Q03164), bromodomain (BRD, residue 1703–1748; UniProt annotations of Q03164), FY-rich N-terminal domain (FYRN, residue 2018–2074; UniProt annotations of Q03164), FY-rich C-terminal domain (FYRC, residue 3666–3747; UniProt annotations of Q03164), taspase1 cleavage site 1/2 (TCS1, residue 2666–2670, D/GADD; TCS2, residue 2718–2722, D/GVDD; the exact cleavage sites are indicated by forward slashes) (Hsieh et al., 2003), transactivator domain (TAD, residue 2829–2883) (Ernst et al., 2001), WDR5 interaction motif (Win; residue 3762–3773) (Patel et al., 2008), and Su(Var)3-9, enhancer-of-zeste, trithorax domain (SET, residue 3829–2945; UniProt annotations of Q03164). The most frequently observed translocation breakpoints (indicated by red arrows) are located in the region between CxxC and PHDs. The three most common MLL-FPs (MLL-AF4, MLL-AF9, and MLL-ENL) are illustrated (the translocation breakpoints and the size of FPs are partially drawn to scale). **(B)** The most frequent MLL rearrangements identified in *MLL*-r leukemia patients. The statistics shown in this figure was obtained from a study of 2,345 *MLL*-r leukemia patients dated from 2003 to 2016 (Meyer et al., 2018). **(C)** Components of MLL-COMPASS. **(D)** MLL-FP (e.g., MLL-AF4, MLL-AF9, or MLL-ENL) in complex with DOT1L and SEC. Figure 1 was created with BioRender.com.

Expression of the developmentally important homeobox (*HOX*) cluster genes is mediated by MLL in normal hematopoietic stem/progenitor cells (HSPCs) (Kawagoe et al., 1999). Genetic knock-out of *Mll* in mice is embryonic lethal, with an altered *Hox* gene pattern, defects in yolk sac hematopoiesis, reduced proliferation and/or survival of hematopoietic progenitors, and defective HSPC activity in the aorta–gonad–mesonephros region (Yu et al., 1995; Hess et al., 1997; Yagi et al., 1998; Ernst et al., 2004). Using conditional *Mll* knock-out (*Mll^–/–^*) mice, McMahon et al. (2007) demonstrated that Mll was not important for the production of mature adult hematopoietic lineages, but it was required for stem cell self-renewal in fetal liver and adult bone marrow. Furthermore, Mll plays an important role in regulating transcription initiation by RNA polymerase II via H3K4 methylation (Wang et al., 2009). Although Mll was shown to influence less than 5% of promoters that carry the H3K4me3 mark in mouse embryonic fibroblasts, these Mll-regulated promoters include important developmental regulators such as the *Hox* genes (Wang et al., 2009). In humans, the *MLL* gene encodes a protein product of 3,969 amino acids (Figure 1A). This product is post-translationally cleaved by threonine aspartase 1 (taspase1) into two distinct modules (MLL-N and MLL-C), then these two modules are assembled together via the FY-rich N- and C-terminal domains (FYRN and FYRC) (García-Alai et al., 2010; Figure 1A). A recent study showed that uncleaved MLL displays higher stability than the assembled dimer (MLL-N/MLL-C) (Zhao et al., 2018). Casein kinase II (CKII) phosphorylates MLL at a location proximal to the taspase1 cleavage site, which facilitates taspase1-dependent processing of MLL into MLL-N and MLL-C (Zhao et al., 2018). This finding suggested that pharmacological targeting of MLL to enhance its stability through inhibition of CKII may present a new therapeutic opportunity in *MLL*-r leukemia, as uncleaved MLL can displace leukemia-causing MLL-fusion proteins (MLL-FPs) from chromatin (Zhao et al., 2018).

MLL-N consists of a Menin-binding motif and a lens epithelium-derived growth factor (LEDGF)-binding domain (Yokoyama and Cleary, 2008); three DNA-binding AT-hook motifs (Zeleznik-Le et al., 1994); two nuclear-localization signals, SNL1 and SNL2 (Yano et al., 1997; Ayton et al., 2004); a pre-CxxC domain (Muntean et al., 2010); a non-methyl-CpG recognizing the CxxC domain (Birke et al., 2002; Ayton et al., 2004; Allen et al., 2006); a post-CxxC domain (Muntean et al., 2010); a bromodomain (BRD); four plant homology domains (PHD fingers) (Fair et al., 2001; Ali et al., 2014); and the homodimerization-facilitating domain FYRN (García-Alai et al., 2010; Figure 1A). The sequences flanking the CxxC domain—the pre-CxxC and post-CxxC domains—were demonstrated to be important for direct interaction of MLL with the polymerase associated factor complex (PAFc) (Muntean et al., 2010). PAFc promotes MLL and MLL-FP recruitment to target loci to activate transcription of target genes such as *HOXA9* (Muntean et al., 2010). Therefore, PAFc is a crucial cofactor for both transcriptional regulation by MLL and leukemogenesis mediated by MLL-FPs (Muntean et al., 2010). The BRD of MLL recognizes acetylated lysine residues, whereas the third PHD finger of MLL specifically interacts with H3K4me2/3 (Chang P.-Y. et al., 2010). Binding of the third PHD finger of MLL to H3K4me3 is required for MLL-dependent gene transcription (Chang P.-Y. et al., 2010).

MLL-C possesses two domains capable of modifying chromatin: a transactivator domain (TAD), followed by a SET [Su(Var)3-9, enhancer-of-zeste, trithorax] domain (Figure 1A). The MLL SET domain confers methyltransferase activity that catalyzes the transfer of a methyl group from S-adenosylmethionine to H3K4 (Milne et al., 2002). MLL-C is further assembled into a larger protein complex that contains several cofactors: WD repeat protein 5 (WDR5), retinoblastoma-binding protein 5 (RBBP5), Set1/Ash2 histone methyltransferase complex subunit ASH2 (ASH2L), and protein dpy-30 homolog (DPY30) (Rao and Dou, 2015). WDR5, RBBP5, ASH2L, and DPY30 form a core entity with the MLL SET domain, and enhance the H3K4 dimethylation activity of the MLL SET domain by ∼600-fold (Dou et al., 2006; Patel et al., 2009). Although complete deletion of the *Mll* gene in mice results in embryonic lethality (Yu et al., 1995), mice that harbor a homozygous SET domain deletion (*Mll*Δ*SET*) survive into adulthood and maintain relatively normal hematopoiesis (Mishra et al., 2014). Because the profile of H3K4 methylation at the *Hoxa* loci remains normal in HSPCs isolated from *Mll*Δ*SET* mice, Mishra et al. (2014) speculated that MLL is not the dominant H3K4 methyltransferase that controls *Hox* gene expression. In addition to MLL, five more MLL family members of H3K4 methyltransferases (MLL2, MLL3, MLL4, SETD1A, and SETD1B) are found in mammals, and they associate with other protein factors to form larger macromolecular complexes called COMPASS (complex of proteins associated with Set1; named for the single yeast homolog) (Rao and Dou, 2015; Li et al., 2016; Slany, 2016; Meeks and Shilatifard, 2017). All of the MLL proteins physically associate with four conserved factors—WDR5, RBBP5, ASH2L, and DPY30 (Figure 1C), which stimulates the H3K4 methyltransferase activity of MLL proteins (Rao and Dou, 2015; Li et al., 2016). Among the six MLL proteins, MLL and MLL2 share two unique factors—Menin and LEDGF (Figure 1C), which mediate the recruitment of MLL/MLL2 to their gene targets (Rao and Dou, 2015). Using mouse embryonic fibroblasts as a cell model, Wang et al. (2009) showed that Menin-interacting Mll and Mll2 are key regulators of *Hox* genes, however, the loss of Mll3/Mll4 had little to no effect on H3K4 methylation of *Hox* loci and the expression of *Hox* genes. This suggests that individual MLL family member may play different functional roles. The MLL TAD interacts with the histone acetyltransferases CBP/p300, MOZ, and MOF, which transfer acetyl groups to H3K27, H3K9, and H4K16, respectively (Slany, 2016). These acetyltransferase activities are associated with *Hox* gene activation by the normal MLL protein (Ernst et al., 2001; Dou et al., 2005; Paggetti et al., 2010).

### Common MLL-Fusion Proteins Associated With *MLL*-r Leukemias

As described above, the presence of *MLL* rearrangements at the 11q23 chromosomal location is associated with poor clinical prognosis, and certain subgroups of *MLL*-r leukemia are associated with worse therapeutic outcomes (Balgobind et al., 2009; Szczepański et al., 2010). As a result of different 11q23 chromosomal translocation events, a total of 135 different *MLL* rearrangements (of which 84 translocations generated in-frame MLL-FPs) were identified in patients with acute leukemia (Meyer et al., 2018). According to the report, the nine most frequent fusion partners of MLL are AF4 (∼36%), AF9 (∼19%), ENL (∼13%), AF10 (∼8%), PTD (∼5%), ELL (∼4%), AF6 (∼4%), EPS15 (∼2%), and AF1Q (∼1%), which together represent more than 92% of the MLL-FPs found in *MLL*-r leukemia patients (Meyer et al., 2018). We will focus on MLL-AF4, MLL-AF9, and MLL-ENL, the three most common MLL-FPs discovered in *MLL*-r leukemia patients (Meyer et al., 2018; Figures 1A,B).

Animal models of human disease are important for studying underlying disease mechanisms and testing therapeutic agents/approaches. Following discovery of numerous MLL-FPs in human patients, biomedical researchers attempted to recapitulate the leukemogenic effects driven by various MLL-FPs in mouse models (Milne, 2017). In various reports, the genetic introduction of MLL-AF4 (Chen et al., 2006; Metzler et al., 2006; Krivtsov et al., 2008; Tamai et al., 2011; Lin et al., 2016), MLL-AF9 (Corral et al., 1996; Dobson et al., 1999; Drynan et al., 2005; Barabé et al., 2007; Krivtsov et al., 2013; Buechele et al., 2015), or MLL-ENL (Lavau et al., 1997; Forster et al., 2003; Drynan et al., 2005; Barabé et al., 2007; Buechele et al., 2015), were demonstrated to be leukemogenic in mice. Further studies revealed the ability of these MLL-FPs to efficiently transform hematopoietic cells at different developmental stages (e.g., hematopoietic stem cells, common myeloid progenitors, and granulocyte and macrophage progenitors) into leukemic cells possessing stem-cell-like properties, such as being capable of self-renewal and leukemia initiation and maintenance (Cozzio et al., 2003; Krivtsov et al., 2006, 2008; Krivtsov and Armstrong, 2007).

### MLL-Rearrangement Links Transcriptional and Epigenetic Abnormalities in Leukemia

MLL-FPs have different chromatin-modifying activities than normal MLL proteins. The C-terminal SET domain of wild-type MLL that harbors H3K4 methyltransferase activity is lost in MLL-FPs (Figure 1). Because multiple MLL fusion partners such as AF4/AF9/ENL/ELL are also components of the super elongation complex [SEC; composed of AF4 (or AFF4), AF9 (or ENL), EAF, ELL, and P-TEFb (positive transcription elongation factor b)] (Luo et al., 2012; Cucinotta and Arndt, 2016), chimeric MLL-FPs recruit the SEC (a crucial regulator of transcriptional elongation) (Figure 1D) and result in aberrant gene expression (Collins and Hess, 2016). Furthermore, several components of the SEC interact with the histone H3K79 methyltransferase DOT1L (disruptor of telomeric silencing 1-like) (Park et al., 2010; Biswas et al., 2011; Shen et al., 2013). Increased levels of H3K79 methylation by DOT1L are found at MLL-FP targeted genomic loci such as *HOXA9* and *MEIS1*, which are associated with leukemic transformation (Okada et al., 2005; Krivtsov et al., 2008; Bernt et al., 2011; Li and Ernst, 2014; Chen et al., 2015; Collins and Hess, 2016). Given that histone H3K79 methylation is associated with active gene transcription (Schübeler et al., 2004), this suggests that DOT1L activity and the altered H3K79 epigenetic signature observed at MLL-FP binding loci may also contribute to the expression of the oncogenic program in *MLL*-r leukemias (Bernt et al., 2011; Jo et al., 2011; Chen et al., 2013; Deshpande et al., 2013). Thus, current evidence suggests that MLL-FPs connect MLL to the SEC and the DOT1L-H3K79 methyltransferase complex (consisting of DOT1L, AF9, AF10, and ENL) (Bernt et al., 2011), thereby contributing to the ectopic expression of the leukemic program (Figure 1D).

## Therapeutic Targeting of the MLL-Fusion Protein Epigenetic Network

**FIGURE 2:**
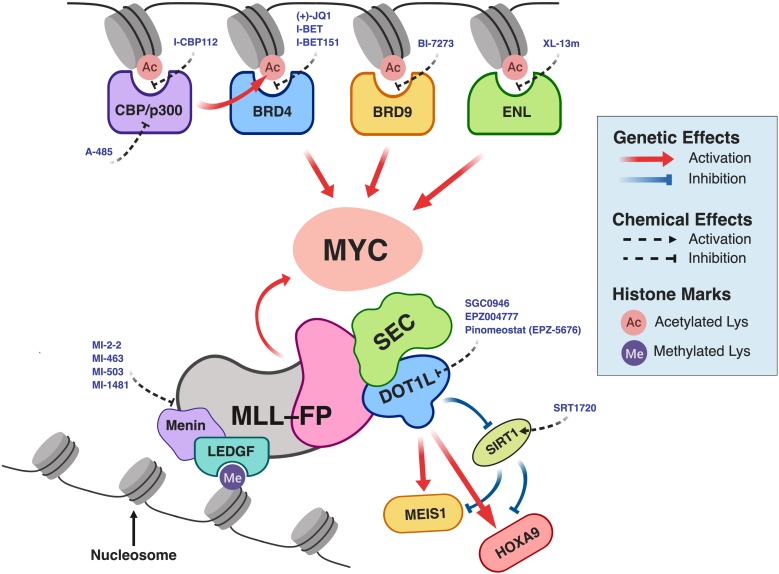
Pharmacological intervention in the MLL-FP epigenetic network in *MLL*-r leukemia. For simplicity, the components of protein complexes interacting with CBP/p300, BRD4, BRD9, or ENL are not illustrated. BRD4 and MLL-FPs (MLL-AF4 or MLL-AF9) together form a complex with SEC and PAFc (Dawson et al., 2011), which directs *MYC* expression. This schematic was created using BioRender.com.

In recent years, chemical inhibitors that target chromatin epigenetic regulators have undergone active development for treatment of various cancers (Filippakopoulos and Knapp, 2014; Shi and Vakoc, 2014; Cai et al., 2015; Fujisawa and Filippakopoulos, 2017; Ribich et al., 2017; Schiedel and Conway, 2018). As discussed above, MLL-FPs associate with cofactors and recruit epigenetic effectors, which eventually lead to aberrant gene expression and leukemic transformation. Therefore, pharmacological disruption of the key proteins in this MLL-FP epigenetic network represents a therapeutic opportunity for the treatments of *MLL*-r leukemias (Figure 2; bottom). Furthermore, *MYC* is a well-known proto-oncogene that is frequently over-expressed in cancer, including in ALL, AML, and *MLL*-r leukemias (Schreiner et al., 2001; Langenau et al., 2003; Luo et al., 2005; Delgado and León, 2010; Dang, 2012; Li L. et al., 2014; Sanchez-Martin and Ferrando, 2017). Indeed, the *MYC* gene is a direct transcriptional target of MLL-FPs (Dawson et al., 2011; Li and Ernst, 2014). Despite its known oncogenic role in *MLL*-r leukemias, direct inhibition of the MYC transcription factor (TF) is challenging, because of its difficult-to-drug three-dimensional structure. Thus, down-regulating *MYC* gene expression by targeting more easily druggable chromatin-binding domain structures on the regulatory proteins that affect *MYC* expression is an active area of research (Figure 2; top). In the following sections, we will discuss the latest approaches to therapeutic targeting of the MLL-FP epigenetic network.

### Targeting Menin–LEDGF–MLL Interactions

Menin, encoded by the *MEN1* gene, acts as a molecular adaptor to tether both MLL and LEDGF and is required for the oncogenic transformation of *MLL*-r leukemia mediated by MLL-FPs (Yokoyama et al., 2005; Yokoyama and Cleary, 2008). Genetic knockout of *Men1* in mouse embryonic fibroblasts demonstrated that Menin is essential for H3K4 trimethylation at *Hox* loci and expression of nearly all *Hox* genes (Wang et al., 2009). Borkin et al. (2015) developed two potent small-molecule inhibitors (MI-463 and MI-503) that block the MLL-binding site on Menin, resulting in down-regulation of MLL-fusion targets (including *Hoxa9 and Meis1* genes), differentiation of leukemic blasts, and prolonged survival of mouse models of *MLL*-r leukemia. A study reported that MLL1 does not require interaction with Menin to sustain hematopoietic stem cell hematopoiesis (Li et al., 2013). This provides a good rational for developing inhibitors of the Menin–MLL interaction to treat *MLL*-r leukemia without affecting normal cells. Indeed, Borkin et al. (2015) revealed that Menin inhibition by MI-463 and MI-503 resulted in no impairment of normal murine hematopoiesis after 10 days of continuous Menin inhibition. Guided by the molecular scaffolds of the two Menin inhibitors, the same group recently reported the development of a more potent Menin inhibitor, MI-1481, which showed low nanomolar inhibition (IC_50_ = 3.6 nM, measured by fluorescence polarization assay using fluorescein-labeled MLL_4__–__43_) against Menin–MLL interactions (Borkin et al., 2018). Compound MI-1481 showed potent activity in cells and *in vivo* models of *MLL*-r leukemias (Borkin et al., 2018).

Lens epithelium-derived growth factor is a transcriptional coactivator that specifically recognizes H3K36me2/3 (histone modifications that are associated with actively transcribed gene loci) through its PWWP domain (Pradeepa et al., 2012; Eidahl et al., 2013; Zhu et al., 2016). Through association with LEDGF, MLL is brought to chromatin to regulate transcription (Yokoyama and Cleary, 2008). MLL-ENL lacking the high-affinity Menin-binding motif (i.e., it cannot interact with Menin efficiently) failed to co-precipitate with endogenous LEDGF, suggesting that LEDGF interacts conjointly with MLL-ENL and Menin, but does not associate with either one of them separately (Yokoyama and Cleary, 2008). Recent reports suggest that LEDGF is dispensable for normal hematopoiesis but important for leukemogenesis; therefore, LEDGF is being considered as a potential drug target for *MLL*-r leukemias (Blokken et al., 2017; Ashkar et al., 2018).

Although wild-type MLL and MLL-FPs share the same MLL N-terminal and retain Menin/LEDGF-binding ability (Figures 1C,D), two studies suggested that MLL and MLL-FPs have different chromatin-tethering mechanisms (Wang et al., 2011; Xu et al., 2016). Wang et al. (2011) reported that MLL-FPs preferentially regulate only a small subset of wild-type MLL target genes using patient-derived leukemic cells and inducible MLL-ENL cellular system. Moreover, Xu et al. (2016) showed by ChIP-seq experiments that MLL and MLL-AF9 localized at different chromatin regions in a murine cell model of MLL-AF9, and they further demonstrated that these differences were due to the ability of MLL C-terminal domain to interact with WDR5. The C-terminal domain of MLL is lost in MLL-FPs, because of 11q23 translocations (Figure 1). These may explain why targeting either Menin or LEDGF could be a therapeutic approach in treating *MLL*-r leukemias, since MLL and MLL-FPs have differential dependence on Menin or LEDGF for activating gene expression.

### Targeting the Enzymatic Core of DOT1L

Disruptor of telomeric silencing 1-like is a methyltransferase that catalyzes H3K79 mono-, di-, and tri-methylation (H3K79me1/2/3), which are associated with active gene expression (Barski et al., 2007; Jo et al., 2011). DOT1L binds to the nucleosomal disk surface and methylates H3K79 located in the globular histone core (Min et al., 2003). Because the aberrant methyltransferase activity of DOT1L is required for the leukemogenesis of *MLL*-r leukemia, DOT1L inhibition is a promising therapeutic intervention (Chang M.-J. et al., 2010; Bernt et al., 2011; Daigle et al., 2011; Nguyen et al., 2011). A number of small-molecule DOT1L inhibitors have been developed in recent years (Anglin and Song, 2013). One inhibitor, pinometostat (EPZ-5676), was identified as a highly potent and selective inhibitor of the DOT1L active site (Daigle et al., 2013). Pinometostat showed moderate to high clearance and low oral bioavailability in a non-clinical pharmacokinetic and metabolic study (Basavapathruni et al., 2014). A phase I clinical study recently revealed that administration of pinometostat was generally safe; however, its efficacy as a single-agent treatment was modest (Stein et al., 2018). Further studies are needed to investigate whether the combination of pinometostat with other pharmacological inhibitors can potentiate its therapeutic activity in patients with *MLL*-r leukemias.

### Targeting Lysine-Specific Demethylase 1 (LSD1)

Lysine-specific demethylase 1 (LSD1 or KDM1A), the first identified histone demethylase (Shi et al., 2004), is an epigenetic eraser of H3K4me1/2 and H3K9me1/2 histone marks (Shi et al., 2004; Forneris et al., 2005; Metzger et al., 2005; Amente et al., 2013). LSD1 is overexpressed in various solid cancers (bladder, breast, lung, and colorectal cancers) (Lim et al., 2010; Hayami et al., 2011), and in AML as well as lymphoid malignancies and myeloproliferative neoplasms (Lokken and Zeleznik-Le, 2012; Schenk et al., 2012; Niebel et al., 2014; Przespolewski and Wang, 2016). Harris et al. (2012) showed that LSD1 is required for sustaining the expression of the MLL-AF9 oncogenic program and identified LSD1 as a therapeutic target for *MLL*-*AF9* leukemia. Genetic knock-down of *Lsd1* by RNAi induced apoptosis and terminal macrophage differentiation of murine *MLL-AF9* cells, and reduced AML leukemia stem cell potential in mice transplanted with murine *MLL-AF9* cells (Harris et al., 2012). Moreover, Harris et al. (2012) found that pharmacological inhibition of LSD1 by OG-86 (also known as Compound B) (Maiques-Diaz and Somervaille, 2016) resulted in inhibition of colony formation and promotion of leukemic cell differentiation in murine *MLL-AF9* cells and primary human *MLL-AF6* and *MLL-AF9* leukemic blasts (Harris et al., 2012). In 2018, ORY-1001 was identified as a highly potent and selective LSD1 covalent inhibitor with good bioavailability after intra-peritoneal and oral administration (Maes et al., 2018). ORY-1001 binds irreversibly and rapidly to the cofactor flavin adenine dinucleotide (FAD) when it is covalently bound to LSD1, but not to free FAD. ORY-1001 showed nanomolar inhibition (IC_50_ = 18 nM) of recombinant LSD1 and low nanomolar activity (EC_50_ < 1 nM in a FACS-based differentiation assay) against cellular LSD1 in THP1 cells (a human *MLL-AF9* cell line) (Maes et al., 2018). ORY-1001 induced blast differentiation in leukemic cell lines (including *MLL*-r leukemic cell lines), primary AML samples, and two AML patients participating in a clinical trial of ORY-1001, and prolonged survival in murine models of acute leukemia (Maes et al., 2018). ORY-1001 is currently under ongoing phase I/IIa study in relapsed or refractory acute leukemia (EUDRACT no. 2013-002447-29) (Maes et al., 2018). Cusan et al. (2018) demonstrated that LSD1 inhibition using GSK-LSD1 (a LSD1 chemical inhibitor) induces an increase in chromatin accessibility on a global level. They also showed that the antileukemic effect exerted by GSK-LSD1 requires the TFs PU.1 and C/EBPα, as the reduction or depletion of PU.1 or C/EBPα expression level, respectively, rendered murine *MLL-AF9* leukemia cells resistant to GSK-LSD1 inhibition (Cusan et al., 2018).

### Down-Regulation of *MYC* by BRD4 Inhibition

Bromodomains are specific epigenetic reader modules that recognize ε-N-acetylation of lysine motifs, the key epigenetic marks for maintaining open chromatin structure, which is associated with transcriptional activation. In the human genome, there are 46 known BRD-containing proteins. Some of these proteins possess more than one BRD, so there are a total of 61 different BRDs found in humans (Filippakopoulos et al., 2012). Large-scale structural and binding studies demonstrated that BRDs do not specifically read a particular acetylated lysine (Kac) sequence, but instead recognize a combination of different Kac motifs (Filippakopoulos et al., 2012). Although BRDs differ in the primary amino acid sequence, they all possess similar structural folds: a left-handed bundle of four α helices (designated αZ, αA, αB, αC) linked by loop regions of various lengths (ZA and BC loops) that contribute to specificity in Kac substrate recognition (Filippakopoulos et al., 2012; Filippakopoulos and Knapp, 2014; Fujisawa and Filippakopoulos, 2017). Large-scale co-crystallization of various Kac peptides and BRDs showed that Kac peptides bind in the central hydrophobic pocket and form hydrogen bonding with an asparagine residue, present in most BRDs (Filippakopoulos et al., 2012; Filippakopoulos and Knapp, 2014; Fujisawa and Filippakopoulos, 2017). Bromodomain-containing protein 4 (BRD4), a member of the bromodomain and extra-terminal domain (BET) family, is a chromatin reader that recognizes Kac residues through two BRDs. It plays key roles in the maintenance of epigenetic memory, cell cycle control, and transcriptional regulation (Dey et al., 2000, 2009; Yang et al., 2005; Devaiah et al., 2012). In 2010, two independent groups discovered the first BRD4 inhibitors, (+)-JQ1 (Filippakopoulos et al., 2010) and I-BET (Nicodeme et al., 2010). These two inhibitors also represent the first examples of small molecules that target epigenetic readers; before the discovery of (+)-JQ1 and I-BET, only epigenetic writers and erasers had been targeted in the field of epigenetics. This discovery opened up a new possibility of treating cancers by targeting epigenetic reader modules.

In Zuber et al. (2011) used an RNA interference (RNAi) genetic screen to identify BRD4 as a therapeutic target in AMLs, including *MLL*-*AF9*-induced AML. They demonstrated that the use of either short-hairpin RNAs (shRNAs) or (+)-JQ1 to induce knock-down or inhibition of Brd4, respectively, led to anti-leukemic effects in cells and *in vivo*. In a murine *MLL-AF9* AML cell model, shRNA or (+)-JQ1 treatments induced cell differentiation and led to depletion of leukemia stem cells and a global reduction in the expression of Myc target genes. Moreover, Zuber’s group used ChIP-qPCR to show that Brd4 occupancy approximately 2 kb upstream of the *Myc* promoter was reduced after exposure to (+)-JQ1. These data suggest that the effects of Brd4 inhibition are linked at least in part to suppression of the Myc-dependent leukemia sustainment program. Indeed, ectopic over-expression of *Myc* cDNA rescued cell cycle arrest and terminal differentiation induced by *Brd4*-targeting shRNAs and (+)-JQ1 treatment. However, over-expression of *Myc* could not rescue cell death induced by (+)-JQ1, suggesting a Myc-independent function of Brd4 in regulating cell survival (Zuber et al., 2011). Consistent with this, another research group employed a large-scale global proteomic strategy to reveal that MLL-FPs that form part of the PAFc and SEC are associated with the epigenetic reader BRD4 (Dawson et al., 2011). Dawson et al. (2011) suggested that BRD4 may function to recruit leukemogenic MLL-FPs (e.g., MLL-AF4 or MLL-AF9) to chromatin for activating expression of oncogenic genes, and therefore they hypothesized that the displacement of BRD4 using chemical inhibitors may have anti-leukemic effect in *MLL*-r leukemias. The authors showed that I-BET151 displaced BRD4 from Kac substrates on the chromatin and resulted in down-regulation of the antiapoptotic gene *BCL2*, cell cycle regulator *CDK6*, and *MYC* (Dawson et al., 2011). Furthermore, I-BET151 showed anti-leukemic activity in cells and *in vivo*, and possessed better pharmacokinetic properties in mice than (+)-JQ1 (Dawson et al., 2011).

Mechanistically, the pronounced inhibition of *MYC* expression by (+)-JQ1 is thought to result from dissociation of BRD4 from MYC super-enhancers (Lovén et al., 2013). Super-enhancers are large clusters of transcriptional enhancers packed with TFs, cofactors, transcription apparatus, and chromatin regulators such as BRD4 (Hnisz et al., 2013). Sustainment of oncogenic *MYC* expression is associated with *MYC* super-enhancers in multiple myeloma (MM1.S) and *MLL*-r leukemia (*MLL*-*AF9*/*Nras^G12D^*) cells (Lovén et al., 2013; Shi et al., 2013; Shi and Vakoc, 2014). BRD4 molecules densely bind to these active super-enhancers and promote transcriptional elongation through physical association with the Mediator coactivator complex and P-TEFb (Lovén et al., 2013; Shi et al., 2013; Xu and Vakoc, 2017). BRD4 recruits and activate P-TEFb, a multi-protein kinase complex that functions to promote transcriptional elongation (Slany, 2016; Xu and Vakoc, 2017). Chemical inhibition of BRD4 by (+)-JQ1 preferentially displaced BRD4 molecules, as well as Mediator and P-TEFb, from the super-enhancers, thereby down-regulating *MYC* and MYC-target gene expression (Lovén et al., 2013).

### Down-Regulation of *MYC* by CBP/p300 Inhibition

Roe et al. (2015) provided evidence that lineage-specific TFs recruit the lysine acetyltransferases CBP or p300 to acetylate the TFs and histone lysine residues at lineage-specific promoters and enhancers in mouse *MLL*-*AF9*/Nras^G12D^ AML cells. BRD4 was shown to bind to hyperacetylated histone residues and acetylated TFs through its BRDs, then promote transcriptional activation via its association with Mediator and P-TEFb. This chromatin-based signaling cascade provides an additional mechanistic explanation for the rapid down-regulation of gene expression by (+)-JQ1, which functions to displace densely localized BRD4 molecules from transcriptionally activated cancer-promoting genes including *BCL2*, *CDK6*, and *MYC* (Roe et al., 2015; Xu and Vakoc, 2017). Given that CBP/p300 play important roles in maintaining *MLL*-r leukemia, this suggests that chemical inhibition of CBP or p300 may present a therapeutic strategy for treating *MLL*-r AMLs (Roe et al., 2015). Small molecules targeting the BRDs of CBP/p300 have been developed (Borah et al., 2011; Hay et al., 2014; Rooney et al., 2014; Brand et al., 2015; Picaud et al., 2015; Schiedel and Conway, 2018). For example, I-CBP112, a potent and selective CBP/p300 inhibitor with modest pharmacokinetic properties, was reported in 2015 (Picaud et al., 2015). I-CBP112 showed inhibitory effects on cancer cell growth in both human and mouse *MLL*-*AF9* AML cell lines and prolonged the survival of mice injected with *MLL*-*AF9* AML cells. Picaud et al. (2015) also reported that I-CBP112 sensitized MOLM-13 *MLL*-r leukemia cells to (+)-JQ1. The combined use of both epigenetic inhibitors could achieve greater antileukemic effects than employing a single agent alone (Picaud et al., 2015). In Lasko et al. (2017), a major breakthrough was achieved in the development of a highly potent, selective, and cell-permeable CBP/p300 inhibitor. Instead of targeting BRDs, the new compound, A-485, binds and inhibits the catalytic core of CBP/p300 and shows inhibitory effects in various cancer cell lines, including MOLM-13 *MLL*-r leukemia cells. Importantly, A-485 was also found to robustly reduce *MYC* expression and show tumor-inhibitory effects in a prostate cancer mouse model.

### Down-Regulation of *MYC* by BRD9 Inhibition

Bromodomain-containing protein 9 (BRD9) is a component of the SWI-SNF chromatin remodeling complex, also known as the BRG1-associated factor (BAF) chromatin remodeling complex in mammals. BRD9 is important for sustaining *MYC* expression *via* its BRD in diverse *MLL*-r leukemia cell lines, including MV4-11, MOLM-13, ML-2, EoL-1, and NOMO-1 (Hohmann et al., 2016). Hohmann et al. (2016) showed that application of RNAi or the small molecule BI-7273 (Martin et al., 2016) to achieve genetic knock-down or chemical inhibition of BRD9, respectively, induced cell growth inhibition and differentiation of *MLL*-r leukemia cell lines through down-regulation of MYC. The same team also devised a domain-swapping strategy to assess the on-target specificity of the chemical probe (BI-7273) on BRD9. In this method, they swapped the BRD of BRD9 with the first BRD of BRD4, to generate a new chimeric protein, BRD9-BET. The full biological function of BRD9 was preserved in BRD9-BET after the domain-swap; however, BI-7273 could no longer inhibit BRD9-BET. This validated the on-target specificity of BI-7273 for the BRD of BRD9.

### Down-Regulation of *MYC* by ENL Inhibition

ENL (eleven-nineteen-leukemia protein or MTTL1) is the third most frequent MLL translocation partner identified in *MLL*-r leukemia patients (Meyer et al., 2018; Figure 1B). ENL is also a component of the SEC, which is observed in the MLL-FP complexes (Zeisig et al., 2005; Yokoyama et al., 2010; Figure 1D). The N-terminal of ENL possesses a YEATS domain. In additional to BRDs recognizing Kac (Dhalluin et al., 1999; Filippakopoulos et al., 2012), in 2014, YEATS domains were discovered to be epigenetic readers of Kac (Li Y. et al., 2014). YEATS domains were named after the five founding members of the YEATS-containing protein family—Yaf9, ENL, AF9, Taf14, and Sas5 (Li Y. et al., 2014). Structurally, the YEATS domains of ENL and AF9 adopt an eight-stranded β-sandwich fold (Li Y. et al., 2014; Wan et al., 2017), which is different from the four-α-helical fold of BRDs (Dhalluin et al., 1999; Filippakopoulos et al., 2012). ENL was found to be important in maintaining AMLs (including *MLL*-r leukemias) by two independent groups in 2017 (Erb et al., 2017; Wan et al., 2017). Depletion of ENL results in down-regulation of key leukemic drivers such as *MYC*, cell growth inhibition, reduced expression of the leukemia stem cell signature, and terminal differentiation of *MLL*-r leukemia cell models (Erb et al., 2017; Wan et al., 2017). Cas9-mediated depletion of ENL prolonged survival in leukemic mouse models, which were xenotransplanted with either MV4-11 or MOLM-13 cells transduced with single-guide RNA targeting the *ENL* gene (Erb et al., 2017; Wan et al., 2017). The two groups also demonstrated that the YEATS reader domain of ENL plays an important role in oncogenic expression and leukemia maintenance through YEATS-Kac interactions. This suggests that pharmacological inhibition of the ENL YEATS domain is a potential therapeutic target for AMLs such as *MLL*-r leukemias (Erb et al., 2017; Wan et al., 2017). Subsequently, another two research groups separately described small molecules capable of inhibiting the YEATS domains of AF9/ENL (Christott et al., 2018; Li et al., 2018). Christott et al. (2018) screened a library of 24,000 compounds using a peptide displacement assay and discovered a small molecule (XS018661) that binds to AF9 and ENL with equilibrium dissociation constant (*K_d_*) values of 523 ± 53 and 745 ± 45 nM, respectively, as determined by isothermal calorimetry (ITC). Li et al. (2018), on the other hand, used structure-guided development to produce selective peptide-based AF9 and ENL inhibitors, termed XL-13a and XL-13m, respectively. They also showed that XL-13m down-regulated key leukemic driver genes including *MYC*, *MYB* (a transcriptional activator), *HOXA9*, and *MEIS1* in MOLM-13 cells (Li et al., 2018).

## Combined Epigenetic Therapies for *MLL*-r Leukemias

Although the DOT1L methyltransferase inhibitor, pinometostat, has cell-inhibitory effect in cells and *in vivo* models of *MLL*-r leukemia (Daigle et al., 2013), recent results from a phase I clinical trial indicated that the small molecule has only modest efficacy in treating *MLL*-r leukemias (Stein et al., 2018). This suggests that combining DOT1L inhibitors with other pharmacological agents may be necessary to boost anti-leukemic efficacy. A genome-wide RNAi screen showed that SIRT1, an NAD^+^-dependent deacetylase, is required to establish a chromatin-repressive state after inhibition of DOT1L (Chen et al., 2015). Consistent with this, a potent activator of SIRT1, SRT1720 (Milne et al., 2007; Mitchell et al., 2014), was demonstrated to synergize with EPZ004777, a DOT1L inhibitor, and enhance anti-proliferative activity against *MLL*-r leukemia cells (Chen et al., 2015). Another study by Gilan et al. (2016) showed that although DOT1L and BRD4 occupy distinct molecular complexes, they functionally cooperate with each other, and that such cooperation is particularly important for highly transcribed genes with proximity to super-enhancers. They found that combined targeting of both DOT1L and BRD4 using SGC0946 (a small-molecule inhibitor of DOT1L) and I-BET, respectively, resulted in growth inhibitory synergy against *MLL*-r cell lines, primary human leukemia cells, and mouse leukemia models (Gilan et al., 2016). In a more recent study, Dafflon et al. (2017) employed an epigenome-focused shRNA library to identify epigenetic regulators that sensitize *MLL*-r leukemia cells treated with EPZ004777. They reported that combination of EPZ004777 and MI-2-2 (an inhibitor of MLL–Menin interaction) enhanced the down-regulation of important leukemia genes (i.e., *MYC*, *HOXA9*, and *MEIS1)* and anti-proliferative effects against *MLL*-r leukemia cells, compared to either single-agent treatment (Dafflon et al., 2017). Maes et al. (2018) recently showed that the LSD1 demethylase inhibitor ORY-1001 could synergize with a retinoid derivative (ATRA), a nucleoside analog [cytosine arabinoside (ARA-C)], a FLT3 inhibitor (quizartinib), DOT1L inhibitors (EPZ-5676; SGC0946), DNMT1 inhibitors (decitabine; azacitidine), an HDAC inhibitor (SAHA), and a BCL2 inhibitor (ABT-737) to inhibit the proliferation of MV4-11 and MOLM-13 *MLL*-r leukemic cell lines.

In advance of identifying the first-in-class YEATS domain inhibitors, scientists used genetic and chemical biology tools to determine if ENL YEATS inhibition synergized with known inhibitors of epigenetic regulators (Erb et al., 2017; Wan et al., 2017). Wan et al. (2017) proved that Cas9-mediated depletion of ENL sensitized MOLM-13 cells to (+)-JQ1 treatment. In a proof-of-concept experiment, Li et al. (2018) used small-molecule chemical probes to demonstrate that the ENL YEATS domain inhibitor XL-13m synergized with either the DOT1L inhibitor pinometostat or the BRD4 inhibitor (+)-JQ1 in reducing the expression of *MYC* in MOLM-13 cells. Erb et al. (2017) applied a novel approach to induce proteasome-mediated acute degradation of ENL using degradation tag (dTAG) technology (Winter et al., 2015; Nabet et al., 2018) in leukemic cells. The dTAG system is a new technology that uses an FKBP12^F36V^-fused target protein-of-interest and a heterobifunctional ligand degrader—a dTAG molecule (e.g., dTAG-13)—that binds FKBP12^F36V^ and cereblon (CRBN) E3 ligase (Winter et al., 2015; Nabet et al., 2018; Mayor-Ruiz and Winter, 2019). This offers a generalized strategy to fuse any target protein to an engineered variant of the immunophilin FKBP12, then tag FKBP12^F36V^-fused target proteins for acute proteasome-mediated degradation. The F36V mutation in FKBP12^F36V^ affords a “bump-hole” strategy that allows specific tagging of the FKBP12^F36V^-fusion proteins, without affecting endogenous FKBP12 proteins. The *FKBP12^F36V^*-target fusion gene can be genetically introduced via transgene expression or CRISPR-mediated locus-specific knock-in Nabet et al. (2018). The dTAG-13 molecule induces dimerization of the FKBP12^F36V^-fused target protein with CRBN E3 ligase, which leads to polyubiquitination of the target protein and proteasomal degradation. After degradation, the dTAG-13 molecules are recycled (i.e., without being degraded) for subsequent rounds of proteasome-mediated degradation of additional FKBP12^F36V^-fused target proteins. Erb et al. (2017) demonstrated that combining dTAG-mediated acute degradation of ENL with pinometostat had additive inhibitory effects on the expression of *Myc* and *Hoxa9* in *MLL*-r cells (MV4-11, *Cas9*^+^, *ENL*-*FKBP12^F36V^*–*HA*^+^, *ENL^–^*^/^*^–^*).

## Concluding Remarks

Chimeric MLL-FPs form complexes with different epigenetic regulators that are capable of rewiring the epigenetic networks and driving the leukemic programs in *MLL*-r leukemias. For example, MLL-FPs recruit DOT1L (an epigenetic writer), and cause aberrant expression of *HOXA9* and *MEIS1*, which leads to leukemic transformation. Moreover, MLL-FPs are able to form a molecular complex with BRD4 (an epigenetic reader), PAFc and SEC to sustain the oncogenic expression of *BCL2*, *CDK6*, and *MYC* in *MLL*-r leukemias (Dawson et al., 2011). Pharmacological intervention with small molecules disrupting the epigenetic networks rewired by the MLL-FPs (Figure 2), holds promises in treating *MLL*-r leukemias. However, in some circumstances, cancer monotherapies using single chemical agents may have sub-optimal efficacy in eliminating cancer cells and are likely limited by the emergence of resistant cell populations (Fong et al., 2015; Rathert et al., 2015; Doroshow et al., 2017). Recent studies have shown that combining epigenetic drug treatments offers improved therapeutic responses over monotherapies in treating various cancers including *MLL*-r leukemias (Doroshow et al., 2017). It is of crucial importance to understand the mechanistic actions of drugs, in order to build a comprehensive rationale for using the drugs in a combinatory setting. This is especially important in targeting context-dependent epigenetic regulatory proteins. The advent of massive parallel sequencing technologies and genetic screening technologies such as RNAi and CRISPR-Cas9 has enabled biomedical scientists to uncover cancer vulnerabilities and discover new therapeutic targets for cancer treatments in a high-throughput manner (Shalem et al., 2015; McDonald et al., 2017; Meyers et al., 2017; Tsherniak et al., 2017). With the emergence of dTAG technology, cancer researchers are equipped with an acute proteasome-mediated protein knock-out method for interrogating biological function and early validation of therapeutic targets, before a valid binding ligand is identified (Winter et al., 2015; Nabet et al., 2018; Mayor-Ruiz and Winter, 2019; Schiedel et al., 2019). With such genetic and chemical discovery tools in hand, we foresee that researchers are empowered to discover and validate important therapeutic targets to treat diseases driven by epigenetic abnormalities such as *MLL*-r leukemias.

## Author Contributions

Both authors listed have made a substantial, direct and intellectual contribution to the work, and approved it for publication.

## Conflict of Interest Statement

The authors declare that the research was conducted in the absence of any commercial or financial relationships that could be construed as a potential conflict of interest.

## References

[B1] Ali M. Hom R. A. Blakeslee W. Ikenouye L. Kutateladze T. G. (2014). Diverse functions of PHD fingers of the MLL/KMT2 subfamily. *Biochim. Biophys. Acta* 1843 366–371. 10.1016/j.bbamcr.2013.11.016 24291127 PMC3925188

[B2] Allen M. D. Grummitt C. G. Hilcenko C. Min S. Tonkin L. M. Johnson C. M. (2006). Solution structure of the nonmethyl-CpG-binding CXXC domain of the leukaemia-associated MLL histone methyltransferase. *EMBO J.* 25 4503–4512. 10.1038/sj.emboj.7601340 16990798 PMC1589984

[B3] Amente S. Lania L. Majello B. (2013). The histone LSD1 demethylase in stemness and cancer transcription programs. *Biochim. Biophys. Acta* 1829 981–986. 10.1016/j.bbagrm.2013.05.002 23684752

[B4] Anglin J. L. Song Y. (2013). A medicinal chemistry perspective for targeting histone H3 lysine-79 methyltransferase DOT1L. *J. Med. Chem.* 56 8972–8983. 10.1021/jm4007752 23879463 PMC4109313

[B5] Arber D. A. Orazi A. Hasserjian R. Thiele J. Borowitz M. J. Beau M. M. (2016). The 2016 revision to the world health organization classification of myeloid neoplasms and acute leukemia. *Blood* 127 2391–2405. 10.1182/blood-2016-03-643544 27069254

[B6] Arrowsmith C. H. Bountra C. Fish P. V. Lee K. Schapira M. (2012). Epigenetic protein families: a new frontier for drug discovery. *Nat. Rev. Drug Discov.* 11 384–400. 10.1038/nrd3674 22498752

[B7] Ashkar S. Schwaller J. Pieters T. Goossens S. Demeulemeester J. Christ F. (2018). LEDGF/p75 is dispensable for hematopoiesis but essential for MLL-rearranged leukemogenesis. *Blood* 131 95–107. 10.1182/blood-2017-05-786962 29084774 PMC5755044

[B8] Ayton P. M. Chen E. H. Cleary M. L. (2004). Binding to nonmethylated CpG DNA is essential for target recognition, transactivation, and myeloid transformation by an MLL oncoprotein. *Mol. Cell. Biol.* 24 10470–10478. 10.1128/mcb.24.23.10470-10478.2004 15542854 PMC529055

[B9] Bach C. Mueller D. Buhl S. Garcia-Cuellar M. P. Slany R. K. (2008). Alterations of the CxxC domain preclude oncogenic activation of mixed-lineage leukemia 2. *Oncogene* 28 815–823. 10.1038/onc.2008.443 19060922

[B10] Balgobind B. V. Raimondi S. C. Harbott J. Zimmermann M. Alonzo T. A. Auvrignon A. (2009). Novel prognostic subgroups in childhood 11q23/*MLL*-rearranged acute myeloid leukemia: results of an international retrospective study. *Blood* 114 2489–2496. 10.1182/blood-2009-04-215152 19528532 PMC2927031

[B11] Bannister A. J. Kouzarides T. (2011). Regulation of chromatin by histone modifications. *Cell Res.* 21 381–395. 10.1038/cr.2011.22 21321607 PMC3193420

[B12] Barabé F. Kennedy J. A. Hope K. J. Dick J. E. (2007). Modeling the initiation and progression of human acute leukemia in mice. *Science* 316 600–604. 10.1126/science.1139851 17463288

[B13] Barski A. Cuddapah S. Cui K. Roh T.-Y. Schones D. E. Wang Z. (2007). High-resolution profiling of histone methylations in the human genome. *Cell* 129 823–837. 10.1016/j.cell.2007.05.009 17512414

[B14] Basavapathruni A. Olhava E. J. Daigle S. R. Therkelsen C. A. Jin L. Boriack-Sjodin A. P. (2014). Nonclinical pharmacokinetics and metabolism of EPZ-5676, a novel DOT1L histone methyltransferase inhibitor. *Biopharm. Drug Dispos.* 35 237–252. 10.1002/bdd.1889 24415392

[B15] Bernt K. M. Armstrong S. A. (2011). Targeting epigenetic programs in *MLL*-rearranged leukemias. *Hematol. Am. Soc. Hematol. Educ. Program* 2011 354–360. 10.1182/asheducation-2011.1.354 22160057

[B16] Bernt K. M. Zhu N. Sinha A. U. Vempati S. Faber J. Krivtsov A. V. (2011). *MLL*-rearranged leukemia is dependent on aberrant H3K79 methylation by DOT1L. *Cancer Cell* 20 66–78. 10.1016/j.ccr.2011.06.010 21741597 PMC3329803

[B17] Birke M. Schreiner S. García-Cuéllar M.-P. Mahr K. Titgemeyer F. Slany R. K. (2002). The MT domain of the proto-oncoprotein MLL binds to CpG-containing DNA and discriminates against methylation. *Nucleic Acids Res.* 30 958–965. 10.1093/nar/30.4.958 11842107 PMC100340

[B18] Biswas D. Milne T. A. Basrur V. Kim J. Elenitoba-Johnson K. S. Allis D. C. (2011). Function of leukemogenic mixed lineage leukemia 1 (MLL) fusion proteins through distinct partner protein complexes. *Proc. Natl. Acad. Sci. U.S.A.* 108 15751–15756. 10.1073/pnas.1111498108 21896721 PMC3179097

[B19] Blokken J. Rijck J. Christ F. Debyser Z. (2017). Protein–protein and protein–chromatin interactions of LEDGF/p75 as novel drug targets. *Drug Discov. Today Technol.* 24 25–31. 10.1016/j.ddtec.2017.11.002 29233296

[B20] Borah J. C. Mujtaba S. Karakikes I. Zeng L. Muller M. Patel J. (2011). A small molecule binding to the coactivator CREB-binding protein blocks apoptosis in cardiomyocytes. *Chem. Biol.* 18 531–541. 10.1016/j.chembiol.2010.12.021 21513889 PMC3103858

[B21] Borkin D. He S. Miao H. Kempinska K. Pollock J. Chase J. (2015). Pharmacologic inhibition of the Menin-MLL interaction blocks progression of MLL leukemia *in vivo*. *Cancer Cell* 27 589–602. 10.1016/j.ccell.2015.02.016 25817203 PMC4415852

[B22] Borkin D. Klossowski S. Pollock J. Miao H. Linhares B. Kempinska K. (2018). Complexity of blocking bivalent protein-protein interactions: development of a highly potent inhibitor of the menin-mixed-lineage leukemia interaction. *J. Med. Chem.* 61 4832–4850. 10.1021/acs.jmedchem.8b00071 29738674 PMC7029623

[B23] Brand M. Measures A. R. Measures A. M. Wilson B. G. Cortopassi W. A. Alexander R. (2015). Small molecule inhibitors of bromodomain–acetyl-lysine interactions. *ACS Chem. Biol.* 10 22–39. 10.1021/cb500996u 25549280

[B24] Broeker P. Super H. Thirman M. Pomykala H. Yonebayashi Y. Tanabe S. (1996). Distribution of 11q23 breakpoints within the *MLL* breakpoint cluster region in *de novo* acute leukemia and in treatment-related acute myeloid leukemia: correlation with scaffold attachment regions and topoisomerase II consensus binding sites. *Blood* 87 1912–1922. 8634439

[B25] Buechele C. Breese E. H. Schneidawind D. Lin C.-H. Jeong J. Duque-Afonso J. (2015). *MLL* leukemia induction by genome editing of human CD34+ hematopoietic cells. *Blood* 126 1683–1694. 10.1182/blood-2015-05-646398 26311362 PMC4591792

[B26] Cai S. F. Chen C.-W. Armstrong S. A. (2015). Drugging chromatin in cancer: recent advances and novel approaches. *Mol. Cell* 60 561–570. 10.1016/j.molcel.2015.10.042 26590715 PMC4701197

[B27] Chang M.-J. Wu H. Achille N. J. Reisenauer M. Chou C.-W. Zeleznik-Le N. J. (2010). Histone H3 lysine 79 methyltransferase Dot1 is required for immortalization by *MLL* oncogenes. *Cancer Res.* 70 10234–10242. 10.1158/0008-5472.can-10-3294 21159644 PMC3040779

[B28] Chang P.-Y. Hom R. A. Musselman C. A. Zhu L. Kuo A. Gozani O. (2010). Binding of the MLL PHD3 finger to histone H3K4me3 is required for MLL-dependent gene transcription. *J. Mol. Biol.* 400 137–144. 10.1016/j.jmb.2010.05.005 20452361 PMC2886590

[B29] Chen C. Sorensen P. Domer P. Reaman G. Korsmeyer S. Heerema N. (1993). Molecular rearrangements on chromosome 11q23 predominate in infant acute lymphoblastic leukemia and are associated with specific biologic variables and poor outcome. *Blood* 81 2386–2393. 8481519

[B30] Chen C.-W. Armstrong S. A. (2015). Targeting DOT1L and *HOX* gene expression in *MLL*-rearranged leukemia and beyond. *Exp. Hematol.* 43 673–684. 10.1016/j.exphem.2015.05.012 26118503 PMC4540610

[B31] Chen C.-W. Koche R. P. Sinha A. U. Deshpande A. J. Zhu N. Eng R. (2015). DOT1L inhibits SIRT1-mediated epigenetic silencing to maintain leukemic gene expression in *MLL*-rearranged leukemia. *Nat. Med.* 21 335–343. 10.1038/nm.3832 25822366 PMC4390532

[B32] Chen L. Deshpande A. Banka D. Bernt K. Dias S. Buske C. (2013). Abrogation of MLL–AF10 and CALM–AF10-mediated transformation through genetic inactivation or pharmacological inhibition of the H3K79 methyltransferase Dot1l. *Leukemia* 27:813. 10.1038/leu.2012.327 23138183 PMC3932800

[B33] Chen W. Li Q. Hudson W. A. Kumar A. Kirchhof N. Kersey J. H. (2006). A murine *Mll-AF4* knock-in model results in lymphoid and myeloid deregulation and hematologic malignancy. *Blood* 108 669–677. 10.1182/blood-2005-08-3498 16551973 PMC1895483

[B34] Chowdhury T. Brady H. (2008). Insights from clinical studies into the role of the *MLL* gene in infant and childhood leukemia. *Blood Cells Mol. Dis.* 40 192–199. 10.1016/j.bcmd.2007.07.005 17905612

[B35] Christott T. Bennett J. Coxon C. Monteiro O. Giroud C. Beke V. (2018). Discovery of a selective inhibitor for the YEATS domains of ENL/AF9. *SLAS Discov* 24 133–141. 10.1177/2472555218809904 30359161

[B36] Collins C. T. Hess J. L. (2016). Deregulation of the HOXA9/MEIS1 axis in acute leukemia. *Curr. Opin. Hematol.* 23 354–361. 10.1097/moh.0000000000000245 27258906 PMC5653247

[B37] Corral J. Lavenir I. Impey H. Warren A. J. Forster A. Larson T. A. (1996). An *Mll–AF9* fusion gene made by homologous recombination causes acute leukemia in chimeric mice: a method to create fusion oncogenes. *Cell* 85 853–861. 10.1016/s0092-8674(00)81269-6 8681380

[B38] Cozzio A. Passegué E. Ayton P. M. Karsunky H. Cleary M. L. Weissman I. L. (2003). Similar MLL-associated leukemias arising from self-renewing stem cells and short-lived myeloid progenitors. *Genes Dev.* 17 3029–3035. 10.1101/gad.1143403 14701873 PMC305255

[B39] Creyghton M. P. Cheng A. W. Welstead G. G. Kooistra T. Carey B. W. Steine E. J. (2010). Histone H3K27ac separates active from poised enhancers and predicts developmental state. *Proc. Natl. Acad. Sci. U.S.A.* 107 21931–21936. 10.1073/pnas.1016071107 21106759 PMC3003124

[B40] Cucinotta C. E. Arndt K. M. (2016). SnapShot: transcription elongation. *Cell* 166:1058.e1. 10.1016/j.cell.2016.07.039 27518568

[B41] Cusan M. Cai S. F. Mohammad H. P. Krivtsov A. Chramiec A. Loizou E. (2018). LSD1 inhibition exerts its antileukemic effect by recommissioning PU.1*- and C/EBPα-dependent enhancers in AML*. *Blood* 131 1730–1742. 10.1182/blood-2017-09-807024 29453291 PMC5897868

[B42] Dafflon C. Craig V. Méreau H. Gräsel J. Engstler S. B. Hoffman G. (2017). Complementary activities of DOT1L and Menin inhibitors in *MLL*-rearranged leukemia. *Leukemia* 31 1269–1277. 10.1038/leu.2016.327 27840424

[B43] Daigle S. R. Olhava E. J. Therkelsen C. A. Basavapathruni A. Jin L. Boriack-Sjodin A. P. (2013). Potent inhibition of DOT1L as treatment of MLL-fusion leukemia. *Blood* 122 1017–1025. 10.1182/blood-2013-04-497644 23801631 PMC3739029

[B44] Daigle S. R. Olhava E. J. Therkelsen C. A. Majer C. R. Sneeringer C. J. Song J. (2011). Selective killing of mixed lineage leukemia cells by a potent small-molecule DOT1L inhibitor. *Cancer Cell* 20 53–65. 10.1016/j.ccr.2011.06.009 21741596 PMC4046888

[B45] Dang C. V. (2012). MYC on the path to cancer. *Cell* 149 22–35. 10.1016/j.cell.2012.03.003 22464321 PMC3345192

[B46] Dawson M. A. Prinjha R. K. Dittmann A. Giotopoulos G. Bantscheff M. Chan W.-I. (2011). Inhibition of BET recruitment to chromatin as an effective treatment for MLL-fusion leukaemia. *Nature* 478:529. 10.1038/nature10509 21964340 PMC3679520

[B47] Delgado D. M. León J. (2010). Myc roles in hematopoiesis and leukemia. *Genes Cancer* 1 605–616. 10.1177/1947601910377495 21779460 PMC3092227

[B48] Deshpande A. J. Chen L. Fazio M. Sinha A. U. Bernt K. M. Banka D. (2013). Leukemic transformation by the *MLL-AF6* fusion oncogene requires the H3K79 methyltransferase Dot1l. *Blood* 121 2533–2541. 10.1182/blood-2012-11-465120 23361907 PMC3612861

[B49] Devaiah B. N. Lewis B. A. Cherman N. Hewitt M. C. Albrecht B. K. Robey P. G. (2012). BRD4 is an atypical kinase that phosphorylates Serine2 of the RNA Polymerase II carboxy-terminal domain. *Proc. Natl. Acad. Sci. U.S.A.* 109 6927–6932. 10.1073/pnas.1120422109 22509028 PMC3345009

[B50] Dey A. Ellenberg J. Farina A. Coleman A. E. Maruyama T. Sciortino S. (2000). A bromodomain protein, MCAP, associates with mitotic chromosomes and fffects G2-to-M transition. *Mol. Cell. Biol.* 20 6537–6549. 10.1128/mcb.20.17.6537-6549.2000 10938129 PMC86127

[B51] Dey A. Nishiyama A. Karpova T. McNally J. Ozato K. (2009). Brd4 marks select genes on mitotic chromatin and directs postmitotic transcription. *Mol. Biol. Cell.* 20 4899–4909. 10.1091/mbc.e09-05-0380 19812244 PMC2785733

[B52] Dhalluin C. Carlson J. E. Zeng L. He C. Aggarwal A. K. Zhou M.-M. (1999). Structure and ligand of a histone acetyltransferase bromodomain. *Nature* 399:491. 10.1038/20974 10365964

[B53] Djabali M. Selleri L. Parry P. Bower M. Young B. D. Evans G. A. (1992). A trithorax–like gene is interrupted by chromosome 11q23 translocations in acute leukaemias. *Nat. Genet.* 2 113–118. 10.1038/ng1092-113 1303259

[B54] Dobson C. L. Warren A. J. Pannell R. Forster A. Lavenir I. Corral J. (1999). The *Mll–AF9* gene fusion in mice controls myeloproliferation and specifies acute myeloid leukaemogenesis. *EMBO J.* 18 3564–3574. 10.1093/emboj/18.13.3564 10393173 PMC1171435

[B55] Döhner H. Weisdorf D. J. Bloomfield C. D. (2015). Acute myeloid leukemia. *N. Engl. J. Med.* 373 1136–1152. 10.1056/nejmra1406184 26376137

[B56] Doroshow D. Eder J. LoRusso P. (2017). BET inhibitors: a novel epigenetic approach. *Ann. Oncol.* 28 1776–1787. 10.1093/annonc/mdx157 28838216

[B57] Dou Y. Milne T. A. Ruthenburg A. J. Lee S. Lee J. Verdine G. L. (2006). Regulation of MLL1 H3K4 methyltransferase activity by its core components. *Nat. Struct. Mol. Biol.* 13:nsmb1128. 10.1038/nsmb1128 16878130

[B58] Dou Y. Milne T. A. Tackett A. J. Smith E. R. Fukuda A. Wysocka J. (2005). Physical association and coordinate function of the H3 K4 methyltransferase MLL1 and the H4 K16 acetyltransferase MOF. *Cell* 121 873–885. 10.1016/j.cell.2005.04.031 15960975

[B59] Drynan L. F. Pannell R. Forster A. Chan N. M. Cano F. Daser A. (2005). *Mll* fusions generated by Cre-*loxP*-mediated *de novo* translocations can induce lineage reassignment in tumorigenesis. *EMBO J.* 24 3136–3146. 10.1038/sj.emboj.7600760 16096649 PMC1201345

[B60] Eidahl J. O. Crowe B. L. North J. A. McKee C. J. Shkriabai N. Feng L. (2013). Structural basis for high-affinity binding of LEDGF PWWP to mononucleosomes. *Nucleic Acids Res.* 41 3924–3936. 10.1093/nar/gkt074 23396443 PMC3616739

[B61] Erb M. A. Scott T. G. Li B. E. Xie H. Paulk J. Seo H.-S. (2017). Transcription control by the ENL YEATS domain in acute leukaemia. *Nature* 543:270. 10.1038/nature21688 28241139 PMC5497220

[B62] Ernst P. Fisher J. K. Avery W. Wade S. Foy D. Korsmeyer S. J. (2004). Definitive hematopoiesis requires the mixed-lineage leukemia gene. *Dev. Cell* 6 437–443. 10.1016/s1534-5807(04)00061-9 15030765

[B63] Ernst P. Wang J. Huang M. Goodman R. H. Korsmeyer S. J. (2001). MLL and CREB bind cooperatively to the nuclear coactivator CREB-binding protein. *Mol. Cell. Biol.* 21 2249–2258. 10.1128/mcb.21.7.2249-2258.2001 11259575 PMC86859

[B64] Fair K. Anderson M. Bulanova E. Mi H. Tropschug M. Diaz M. O. (2001). Protein interactions of the MLL PHD fingers modulate MLL target gene regulation in human cells. *Mol. Cell. Biol.* 21 3589–3597. 10.1128/mcb.21.10.3589-3597.2001 11313484 PMC100280

[B65] Felix C. A. (1998). Secondary leukemias induced by topoisomerase-targeted drugs. *Biochim. Biophys. Acta* 1400 233–255. 10.1016/s0167-4781(98)00139-0 9748598

[B66] Filippakopoulos P. Knapp S. (2014). Targeting bromodomains: epigenetic readers of lysine acetylation. *Nat. Rev. Drug Discov.* 13 337–356. 10.1038/nrd4286 24751816

[B67] Filippakopoulos P. Picaud S. Mangos M. Keates T. Lambert J.-P. Barsyte-Lovejoy D. (2012). Histone recognition and large-scale structural analysis of the human bromodomain family. *Cell* 149 214–231. 10.1016/j.cell.2012.02.013 22464331 PMC3326523

[B68] Filippakopoulos P. Qi J. Picaud S. Shen Y. Smith W. B. Fedorov O. (2010). Selective inhibition of BET bromodomains. *Nature* 468 1067–1073. 10.1038/nature09504 20871596 PMC3010259

[B69] Fong C. Gilan O. Lam E. Y. Rubin A. F. Ftouni S. Tyler D. (2015). BET inhibitor resistance emerges from leukaemia stem cells. *Nature* 525 538–542. 10.1038/nature14888 26367796 PMC6069604

[B70] Forneris F. Binda C. Vanoni M. Battaglioli E. Mattevi A. (2005). Human histone demethylase LSD1 reads the histone code. *J. Biol. Chem.* 280 41360–41365. 10.1074/jbc.m509549200 16223729

[B71] Forster A. Pannell R. Drynan L. F. McCormack M. Collins E. C. Daser A. (2003). Engineering de novo reciprocal chromosomal translocations associated with *Mll* to replicate primary events of human cancer. *Cancer Cell* 3 449–458. 10.1016/s1535-6108(03)00106-5 12781363

[B72] Fujisawa T. Filippakopoulos P. (2017). Functions of bromodomain-containing proteins and their roles in homeostasis and cancer. *Nat. Rev. Mol. Cell Biol.* 18 246–262. 10.1038/nrm.2016.143 28053347

[B73] Gagnon G. Childs C. LeMaistre A. Keating M. Cork A. Trujillo J. (1989). Molecular heterogeneity in acute leukemia lineage switch. *Blood* 74 2088–2095. 2553159

[B74] García-Alai M. M. Allen M. D. Joerger A. C. Bycroft M. (2010). The structure of the FYR domain of transforming growth factor beta regulator 1. *Protein Sci.* 19 1432–1438. 10.1002/pro.404 20506279 PMC2970912

[B75] Gilan O. Lam E. Y. Becher I. Lugo D. Cannizzaro E. Joberty G. (2016). Functional interdependence of BRD4 and DOT1L in MLL leukemia. *Nat. Struct. Mol. Biol.* 23 673–681. 10.1038/nsmb.3249 27294782

[B76] Greaves M. (1997). Aetiology of acute leukaemia. *Lancet* 349 344–349. 10.1016/s0140-6736(96)09412-3 9024390

[B77] Gu Y. Nakamura T. Alder H. Prasad R. Canaani O. Cimino G. (1992). The t(4;11) chromosome translocation of human acute leukemias fuses the *ALL-1* gene, related to Drosophila *trithorax*, to the *AF-4* gene. *Cell* 71 701–708. 10.1016/0092-8674(92)90603-a 1423625

[B78] Harris W. J. Huang X. Lynch J. T. Spencer G. J. Hitchin J. R. Li Y. (2012). The histone demethylase KDM1A sustains the oncogenic potential of MLL-AF9 leukemia stem cells. *Cancer Cell* 21 473–487. 10.1016/j.ccr.2012.03.014 22464800

[B79] Hawkins D. R. Hon G. C. Yang C. Antosiewicz-Bourget J. E. Lee L. K. Ngo Q.-M. (2011). Dynamic chromatin states in human ES cells reveal potential regulatory sequences and genes involved in pluripotency. *Cell Res.* 21 1393–1409. 10.1038/cr.2011.146 21876557 PMC3193447

[B80] Hay D. A. Fedorov O. Martin S. Singleton D. C. Tallant C. Wells C. (2014). Discovery and optimization of small-molecule ligands for the CBP/p300 bromodomains. *J. Am. Chem. Soc.* 136 9308–9319. 10.1021/ja412434f 24946055 PMC4183655

[B81] Hayami S. Kelly J. D. Cho H. Yoshimatsu M. Unoki M. Tsunoda T. (2011). Overexpression of LSD1 contributes to human carcinogenesis through chromatin regulation in various cancers. *Int. J. Cancer* 128 574–586. 10.1002/ijc.25349 20333681

[B82] Hayashi Y. Sugita K. Nakazawa S. Abe T. Kojima S. Inaba T. (1990). Karyotypic patterns in acute mixed lineage leukemia. *Leukemia* 4 121–126. 2137547

[B83] Heerema N. Sather H. Ge J. Arthur D. Hilden J. Trigg M. (1999). Cytogenetic studies of infant acute lymphoblastic leukemia: poor prognosis of infants with t(4;11) – a report of the children’s cancer group. *Leukemia* 13 679–686. 10.1038/sj.leu.2401413 10374870

[B84] Hess J. Yu B. Li B. Hanson R. Korsmeyer S. (1997). Defects in yolk sac hematopoiesis in *Mll*-null embryos. *Blood* 90 1799–1806. 9292512

[B85] Hezroni H. Sailaja B. Meshorer E. (2011). Pluripotency-related, valproic acid (VPA)-induced genome-wide histone H3 lysine 9 (H3K9) acetylation patterns in embryonic stem cells. *J. Biol. Chem.* 286 35977–35988. 10.1074/jbc.m111.266254 21849501 PMC3195619

[B86] Hilden J. Smith F. Frestedt J. McGlennen R. Howells W. Sorensen P. (1997). *MLL* gene rearrangement, cytogenetic 11q23 abnormalities, and expression of the NG2 molecule in infant acute myeloid leukemia. *Blood* 89 3801–3805. 9160687

[B87] Hilden J. M. Dinndorf P. A. Meerbaum S. O. Sather H. Villaluna D. Heerema N. A. (2006). Analysis of prognostic factors of acute lymphoblastic leukemia in infants: report on CCG 1953 from the children’s oncology group. *Blood* 108 441–451. 10.1182/blood-2005-07-3011 16556894 PMC1895499

[B88] Hnisz D. Abraham B. J. Lee T. Lau A. Saint-André V. Sigova A. A. (2013). Super-enhancers in the control of cell identity and disease. *Cell* 155 934–947. 10.1016/j.cell.2013.09.053 24119843 PMC3841062

[B89] Hohmann A. F. Martin L. J. Minder J. L. Roe J.-S. Shi J. Steurer S. (2016). Sensitivity and engineered resistance of myeloid leukemia cells to BRD9 inhibition. *Nat. Chem. Biol.* 12 672–679. 10.1038/nchembio.2115 27376689 PMC4990482

[B90] Hsieh J. Cheng E. Korsmeyer S. J. (2003). Taspase1: a threonine aspartase required for cleavage of MLL and proper *HOX* gene expression. *Cell* 115 293–303. 10.1016/s0092-8674(03)00816-x 14636557

[B91] Inaba H. Greaves M. Mullighan C. G. (2013). Acute lymphoblastic leukaemia. *Lancet* 381 1943–1955. 10.1016/s0140-6736(12)62187-4 23523389 PMC3816716

[B92] Jo S. Y. Granowicz E. M. Maillard I. Thomas D. Hess J. L. (2011). Requirement for Dot1l in murine postnatal hematopoiesis and leukemogenesis by *MLL* translocation. *Blood* 117 4759–4768. 10.1182/blood-2010-12-327668 21398221 PMC3100687

[B93] Kawagoe H. Humphries R. Blair A. Sutherland H. Hogge D. (1999). Expression of *HOX* genes, *HOX* cofactors, and *MLL* in phenotypically and functionally defined subpopulations of leukemic and normal human hematopoietic cells. *Leukemia* 13 687–698. 10.1038/sj.leu.2401410 10374871

[B94] Krejčí J. Uhlířová R. Galiová G. Kozubek S. Šmigová J. Bártová E. (2009). Genome-wide reduction in H3K9 acetylation during human embryonic stem cell differentiation. *J. Cell Physiol.* 219 677–687. 10.1002/jcp.21714 19202556

[B95] Krivtsov A. V. Armstrong S. A. (2007). *MLL* translocations, histone modifications and leukaemia stem-cell development. *Nat. Rev. Cancer* 7 nrc2253. 10.1038/nrc2253 17957188

[B96] Krivtsov A. V. Feng Z. Lemieux M. E. Faber J. Vempati S. Sinha A. U. (2008). H3K79 methylation profiles define murine and human MLL-AF4 leukemias. *Cancer Cell* 14 355–368. 10.1016/j.ccr.2008.10.001 18977325 PMC2591932

[B97] Krivtsov A. V. Figueroa M. Sinha A. Stubbs M. Feng Z. Valk P. (2013). Cell of origin determines clinically relevant subtypes of *MLL*-rearranged AML. *Leukemia* 27 852–860. 10.1038/leu.2012.363 23235717 PMC4693300

[B98] Krivtsov A. V. Hoshii T. Armstrong S. A. (2017). Mixed-lineage leukemia fusions and chromatin in leukemia. *Cold Spring Harb. Perspect. Med.* 7 a026658. 10.1101/cshperspect.a026658 28242784 PMC5666623

[B99] Krivtsov A. V. Twomey D. Feng Z. Stubbs M. C. Wang Y. Faber J. (2006). Transformation from committed progenitor to leukaemia stem cell initiated by MLL–AF9. *Nature* 442 818–822. 10.1038/nature04980 16862118

[B100] Langenau D. M. Traver D. Ferrando A. A. Kutok J. L. Aster J. C. Kanki J. P. (2003). Myc-induced T cell leukemia in transgenic zebrafish. *Science* 299 887–890. 10.1126/science.1080280 12574629

[B101] Lasko L. M. Jakob C. G. Edalji R. P. Qiu W. Montgomery D. Digiammarino E. L. (2017). Discovery of a selective catalytic p300/CBP inhibitor that targets lineage-specific tumours. *Nature* 550 128–132. 10.1038/nature24028 28953875 PMC6050590

[B102] Lavau C. Szilvassy S. J. Slany R. Cleary M. L. (1997). Immortalization and leukemic transformation of a myelomonocytic precursor by retrovirally transduced HRX–ENL. *EMBO J.* 16 4226–4237. 10.1093/emboj/16.14.4226 9250666 PMC1170048

[B103] Li B. E. Ernst P. (2014). Two decades of leukemia oncoprotein epistasis: the *MLL1* paradigm for epigenetic deregulation in leukemia. *Exp. Hematol.* 42 995–1012. 10.1016/j.exphem.2014.09.006 25264566 PMC4307938

[B104] Li B. E. Gan T. Meyerson M. Rabbitts T. H. Ernst P. (2013). Distinct pathways regulated by menin and by MLL1 in hematopoietic stem cells and developing B cells. *Blood* 122 2039–2046. 10.1182/blood-2013-03-486647 23908472 PMC3778547

[B105] Li L. Osdal T. Ho Y. Chun S. Nald T. Agarwal P. (2014). SIRT1 activation by a c-MYC oncogenic network promotes the maintenance and drug resistance of human FLT3-ITD acute myeloid leukemia stem cells. *Cell Stem Cell* 15 431–446. 10.1016/j.stem.2014.08.001 25280219 PMC4305398

[B106] Li Y. Wen H. Xi Y. Tanaka K. Wang H. Peng D. (2014). AF9 YEATS domain links histone acetylation to DOT1L-mediated H3K79 methylation. *Cell* 159 558–571. 10.1016/j.cell.2014.09.049 25417107 PMC4344132

[B107] Li X. Li X.-M. Jiang Y. Liu Z. Cui Y. Fung K. (2018). Structure-guided development of YEATS domain inhibitors by targeting π-π-π stacking. *Nat. Chem. Biol.* 14 1140–1149. 10.1038/s41589-018-0144-y 30374167 PMC6503841

[B108] Li Y. Han J. Zhang Y. Cao F. Liu Z. Li S. (2016). Structural basis for activity regulation of MLL family methyltransferases. *Nature* 530 447–452. 10.1038/nature16952 26886794 PMC5125619

[B109] Lim S. Janzer A. Becker A. Zimmer A. Schüle R. Buettner R. (2010). Lysine-specific demethylase 1 (LSD1) is highly expressed in ER-negative breast cancers and a biomarker predicting aggressive biology. *Carcinogenesis* 31 512–520. 10.1093/carcin/bgp324 20042638

[B110] Lin S. Luo R. T. Ptasinska A. Kerry J. Assi S. A. Wunderlich M. (2016). Instructive role of MLL-fusion proteins revealed by a model of t(4;11) pro-B acute lymphoblastic leukemia. *Cancer Cell* 30 737–749. 10.1016/j.ccell.2016.10.008 27846391

[B111] Lokken A. A. Zeleznik-Le N. J. (2012). Breaking the LSD1/KDM1A addiction: therapeutic targeting of the epigenetic modifier in AML. *Cancer Cell* 21 451–453. 10.1016/j.ccr.2012.03.027 22516254 PMC3996681

[B112] Lovén J. Hoke H. A. Lin C. Y. Lau A. Orlando D. A. Vakoc C. R. (2013). Selective inhibition of tumor oncogenes by disruption of super-enhancers. *Cell* 153 320–334. 10.1016/j.cell.2013.03.036 23582323 PMC3760967

[B113] Luo H. Li Q. O’Neal J. Kreisel F. Beau M. M. Tomasson M. H. (2005). c-Myc rapidly induces acute myeloid leukemia in mice without evidence of lymphoma-associated antiapoptotic mutations. *Blood* 106 2452–2461. 10.1182/blood-2005-02-0734 15972450

[B114] Luo Z. Lin C. Shilatifard A. (2012). The super elongation complex (SEC) family in transcriptional control. *Nat. Rev. Mol. Cell Biol* 13 543–547. 10.1038/nrm3417 22895430

[B115] Maes T. Mascaró C. Tirapu I. Estiarte A. Ciceri F. Lunardi S. (2018). ORY-1001, a potent and selective covalent KDM1A inhibitor, for the treatment of acute leukemia. *Cancer Cell* 33 495–511.e12. 10.1016/j.ccell.2018.02.002 29502954

[B116] Maiques-Diaz A. Somervaille T. C. (2016). LSD1: biologic roles and therapeutic targeting. *Epigenomics* 8 1103–1116. 10.2217/epi-2016-0009 27479862 PMC5066116

[B117] Marchesi F. Girardi K. Avvisati G. (2011). Pathogenetic, clinical, and prognostic features of adult t(4;11)(q21;q23)/*MLL-AF4* positive B-cell acute lymphoblastic leukemia. *Adv. Hematol.* 2011:621627. 10.1155/2011/621627 22190943 PMC3235494

[B118] Martin L. J. Koegl M. Bader G. Cockcroft X.-L. Fedorov O. Fiegen D. (2016). Structure-based design of an *in vivo* active selective BRD9 inhibitor. *J. Med. Chem.* 59 4462–4475. 10.1021/acs.jmedchem.5b01865 26914985 PMC4885110

[B119] Martinez-Climent J. Thirman M. Espinosa R. Beau L. M. Rowley J. (1995). Detection of 11q23/*MLL* rearrangements in infant leukemias with fluorescence in situ hybridization and molecular analysis. *Leukemia* 9 1299–1304. 7643616

[B120] Mayor-Ruiz C. Winter G. E. (2019). Identification and characterization of cancer vulnerabilities *via* targeted protein degradation. *Drug Discov. Today Technol.* 10.1016/j.ddtec.2018.12.003 31200863

[B121] McDonald R. E. de Weck A. Schlabach M. R. Billy E. Mavrakis K. J. Hoffman G. R. (2017). Project DRIVE: a compendium of cancer dependencies and synthetic lethal relationships uncovered by large-scale, deep RNAi screening. *Cell* 170 577–592.e10. 10.1016/j.cell.2017.07.005 28753431

[B122] McMahon K. A. Hiew S. Hadjur S. Veiga-Fernandes H. Menzel U. Price A. J. (2007). *Mll* has a critical role in fetal and adult hematopoietic stem cell self-renewal. *Cell Stem Cell* 1 338–345. 10.1016/j.stem.2007.07.002 18371367

[B123] Meeks J. J. Shilatifard A. (2017). Multiple roles for the MLL/COMPASS family in the epigenetic regulation of gene expression and in cancer. *Annu. Rev. Cancer Biol.* 1 425–446. 10.1146/annurev-cancerbio-050216-034333

[B124] Metzger E. Wissmann M. Yin N. Müller J. M. Schneider R. Peters A. H. (2005). LSD1 demethylates repressive histone marks to promote androgen-receptor-dependent transcription. *Nature* 437 436–439. 10.1038/nature04020 16079795

[B125] Metzler M. Forster A. Pannell R. Arends M. Daser A. Lobato M. (2006). A conditional model of MLL-AF4 B-cell tumourigenesis using invertor technology. *Oncogene* 25 3093–3103. 10.1038/sj.onc.1209636 16607274

[B126] Meyer C. Burmeister T. Gröger D. Tsaur G. Fechina L. Renneville A. (2018). The *MLL* recombinome of acute leukemias in 2017. *Leukemia* 32 273–284. 10.1038/leu.2017.213 28701730 PMC5808070

[B127] Meyers R. M. Bryan J. G. McFarland J. M. Weir B. A. Sizemore A. E. Xu H. (2017). Computational correction of copy number effect improves specificity of CRISPR–Cas9 essentiality screens in cancer cells. *Nat. Genet.* 49 1779–1784. 10.1038/ng.3984 29083409 PMC5709193

[B128] Milne J. C. Lambert P. D. Schenk S. Carney D. P. Smith J. J. Gagne D. J. (2007). Small molecule activators of SIRT1 as therapeutics for the treatment of type 2 diabetes. *Nature* 450 712–716. 10.1038/nature06261 18046409 PMC2753457

[B129] Milne T. A. (2017). Mouse models of MLL leukemia: recapitulating the human disease. *Blood* 129 2217–2223. 10.1182/blood-2016-10-691428 28179274 PMC5399479

[B130] Milne T. A. Briggs S. D. Brock H. W. Martin M. Gibbs D. Allis C. D. (2002). MLL targets SET domain methyltransferase activity to *Hox* gene promoters. *Mol. Cell* 10 1107–1117. 10.1016/s1097-2765(02)00741-4 12453418

[B131] Min J. Feng Q. Li Z. Zhang Y. Xu R.-M. (2003). Structure of the catalytic domain of human DOT1L, a non-SET domain nucleosomal histone methyltransferase. *Cell* 112 711–723. 10.1016/s0092-8674(03)00114-4 12628190

[B132] Mirro J. Zipf T. Pui C. Kitchingman G. Williams D. Melvin S. (1985). Acute mixed lineage leukemia: clinicopathologic correlations and prognostic significance. *Blood* 66 1115–1123. 3931724

[B133] Mishra B. P. Zaffuto K. M. Artinger E. L. Org T. Mikkola H. Cheng C. (2014). The histone methyltransferase activity of MLL1 is dispensable for hematopoiesis and leukemogenesis. *Cell Rep.* 7 1239–1247. 10.1016/j.celrep.2014.04.015 24813891 PMC4120120

[B134] Mitchell S. J. Martin-Montalvo A. Mercken E. M. Palacios H. H. Ward T. M. Abulwerdi G. (2014). The SIRT1 activator SRT1720 extends lifespan and improves health of mice fed a standard diet. *Cell Rep.* 6 836–843. 10.1016/j.celrep.2014.01.031 24582957 PMC4010117

[B135] Mullighan C. G. Goorha S. Radtke I. Miller C. B. Coustan-Smith E. Dalton J. D. (2007). Genome-wide analysis of genetic alterations in acute lymphoblastic leukaemia. *Nature* 446 758–764. 10.1038/nature05690 17344859

[B136] Muntean A. G. Hess J. L. (2012). The pathogenesis of mixed-lineage leukemia. *Annu. Rev. Pathol. Mech. Dis.* 7 283–301. 10.1146/annurev-pathol-011811-132434 22017583 PMC3381338

[B137] Muntean A. G. Tan J. Sitwala K. Huang Y. Bronstein J. Connelly J. A. (2010). The PAF complex synergizes with MLL fusion proteins at *HOX* loci to promote leukemogenesis. *Cancer Cell* 17 609–621. 10.1016/j.ccr.2010.04.012 20541477 PMC2888888

[B138] Musselman C. A. Lalonde M.-E. Côté J. Kutateladze T. G. (2012). Perceiving the epigenetic landscape through histone readers. *Nat. Struct. Mol. Biol.* 19 1218–1227. 10.1038/nsmb.2436 23211769 PMC3645987

[B139] Nabet B. Roberts J. M. Buckley D. L. Paulk J. Dastjerdi S. Yang A. (2018). The dTAG system for immediate and target-specific protein degradation. *Nat. Chem. Biol.* 14 431–441. 10.1038/s41589-018-0021-8 29581585 PMC6295913

[B140] Nguyen A. Taranova O. He J. Zhang Y. (2011). DOT1L, the H3K79 methyltransferase, is required for MLL-AF9–mediated leukemogenesis. *Blood* 117 6912–6922. 10.1182/blood-2011-02-334359 21521783 PMC3128482

[B141] Nicodeme E. Jeffrey K. L. Schaefer U. Beinke S. Dewell S. Chung C. (2010). Suppression of inflammation by a synthetic histone mimic. *Nature* 468 1119–1123. 10.1038/nature09589 21068722 PMC5415086

[B142] Niebel D. Kirfel J. Janzen V. Höller T. Majores M. Gütgemann I. (2014). Lysine-specific demethylase 1 (LSD1) in hematopoietic and lymphoid neoplasms. *Blood* 124 151–152. 10.1182/blood-2014-04-569525 24993879

[B143] Okada Y. Feng Q. Lin Y. Jiang Q. Li Y. Coffield V. M. (2005). hDOT1L links histone methylation to leukemogenesis. *Cell* 121 167–178. 10.1016/j.cell.2005.02.020 15851025

[B144] Paggetti J. Largeot A. Aucagne R. Jacquel A. Lagrange B. Yang X.-J. (2010). Crosstalk between leukemia-associated proteins MOZ and MLL regulates *HOX* gene expression in human cord blood CD34+ cells. *Oncogene* 29 5019–5031. 10.1038/onc.2010.254 20581860

[B145] Papaemmanuil E. Gerstung M. Bullinger L. Gaidzik V. I. Paschka P. Roberts N. D. (2016). Genomic classification and prognosis in acute myeloid leukemia. *N. Engl. J. Med.* 374 2209–2221. 10.1056/nejmoa1516192 27276561 PMC4979995

[B146] Park G. Gong Z. Chen J. Kim J.-E. (2010). Characterization of the DOT1L network: implications of diverse roles for DOT1L. *Protein J.* 29 213–223. 10.1007/s10930-010-9242-8 20431927

[B147] Patel A. Dharmarajan V. Cosgrove M. S. (2008). Structure of WDR5 bound to mixed lineage leukemia protein-1 peptide. *J. Biol. Chem.* 283 32158–32161. 10.1074/jbc.c800164200 18829459

[B148] Patel A. Dharmarajan V. Vought V. E. Cosgrove M. S. (2009). On the mechanism of multiple lysine methylation by the human mixed lineage leukemia protein-1 (MLL1) core complex. *J. Biol. Chem.* 284 24242–24256. 10.1074/jbc.m109.014498 19556245 PMC2782018

[B149] Picaud S. Fedorov O. Thanasopoulou A. Leonards K. Jones K. Meier J. (2015). Generation of a selective small molecule inhibitor of the CBP/p300 bromodomain for leukemia therapy. *Cancer Res.* 75 5106–5119. 10.1158/0008-5472.can-15-0236 26552700 PMC4948672

[B150] Pieters R. Schrappe M. Lorenzo P. Hann I. Rossi G. Felice M. (2007). A treatment protocol for infants younger than 1 year with acute lymphoblastic leukaemia (Interfant-99): an observational study and a multicentre randomised trial. *Lancet* 370 240–250. 10.1016/s0140-6736(07)61126-x 17658395

[B151] Pradeepa M. M. Sutherland H. G. Ule J. Grimes G. R. Bickmore W. A. (2012). Psip1/Ledgf p52 binds methylated histone H3K36 and splicing factors and contributes to the regulation of alternative splicing. *Plos. Genet.* 8:e1002717. 10.1371/journal.pgen.1002717 22615581 PMC3355077

[B152] Przespolewski A. Wang E. S. (2016). Inhibitors of LSD1 as a potential therapy for acute myeloid leukemia. *Expert Opin. Investig. Drug* 25 771–780. 10.1080/13543784.2016.1175432 27077938

[B153] Radtke I. Mullighan C. G. Ishii M. Su X. Cheng J. Ma J. (2009). Genomic analysis reveals few genetic alterations in pediatric acute myeloid leukemia. *Proc. Natl. Acad. Sci. U.S.A.* 106 12944–12949. 10.1073/pnas.0903142106 19651601 PMC2716382

[B154] Rao R. C. Dou Y. (2015). Hijacked in cancer: the KMT2 (MLL) family of methyltransferases. *Nat. Rev. Cancer* 15 334–346. 10.1038/nrc3929 25998713 PMC4493861

[B155] Rathert P. Roth M. Neumann T. Muerdter F. Roe J.-S. Muhar M. (2015). Transcriptional plasticity promotes primary and acquired resistance to BET inhibition. *Nature* 525 543–547. 10.1038/nature14898 26367798 PMC4921058

[B156] Ribich S. Harvey D. Copeland R. A. (2017). Drug discovery and chemical biology of cancer epigenetics. *Cell Chem. Biol.* 24 1120–1147. 10.1016/j.chembiol.2017.08.020 28938089

[B157] Roberts K. G. Mullighan C. G. (2015). Genomics in acute lymphoblastic leukaemia: insights and treatment implications. *Nat. Rev. Clin. Oncol.* 12 344–357. 10.1038/nrclinonc.2015.38 25781572

[B158] Roe J.-S. Mercan F. Rivera K. Pappin D. J. Vakoc C. R. (2015). BET bromodomain inhibition suppresses the function of hematopoietic transcription factors in acute myeloid leukemia. *Mol. Cell* 58 1028–1039. 10.1016/j.molcel.2015.04.011 25982114 PMC4475489

[B159] Rooney T. P. Filippakopoulos P. Fedorov O. Picaud S. Cortopassi W. A. Hay D. A. (2014). A series of potent CREBBP bromodomain ligands reveals an induced-fit pocket stabilized by a cation–π interaction. *Angew. Chem. Int. Ed. Engl.* 53 6126–6130. 10.1002/anie.201402750 24821300 PMC4298791

[B160] Rowley J. (1993). Rearrangements involving chromosome band 11Q23 in acute leukaemia. *Semin. Cancer Biol.* 4 377–385. 8142623

[B161] Sanchez-Martin M. Ferrando A. (2017). The NOTCH1-MYC highway toward T-cell acute lymphoblastic leukemia. *Blood* 129 1124–1133. 10.1182/blood-2016-09-692582 28115368

[B162] Satake N. Maseki N. Nishiyama M. Kobayashi H. Sakurai M. Inaba H. (1999). Chromosome abnormalities and *MLL* rearrangements in acute myeloid leukemia of infants. *Leukemia* 13 1013–1017. 10.1038/sj.leu.2401439 10400416

[B163] Schenk T. Chen W. Göllner S. Howell L. Jin L. Hebestreit K. (2012). Inhibition of the LSD1 (KDM1A) demethylase reactivates the all-trans-retinoic acid differentiation pathway in acute myeloid leukemia. *Nat. Med.* 18 605–611. 10.1038/nm.2661 22406747 PMC3539284

[B164] Schiedel M. Conway S. J. (2018). Small molecules as tools to study the chemical epigenetics of lysine acetylation. *Curr. Opin. Chem. Biol.* 45 166–178. 10.1016/j.cbpa.2018.06.015 29958150

[B165] Schiedel M. Moroglu M. Ascough D. M. Chamberlain A. E. Kamps J. J. Sekirnik A. R. (2019). Chemical epigenetics: the impact of chemical- and chemical biology techniques on bromodomain target validation. *Angew. Chem. Int. Ed. Engl.* [Epub ahead of print]. 10.1002/anie.201812164 30633431

[B166] Schreiner S. Birke M. García-Cuéllar M. Zilles O. Greil J. Slany R. (2001). MLL-ENL causes a reversible and *myc*-dependent block of myelomonocytic cell differentiation. *Cancer Res.* 61 6480–6486. 11522644

[B167] Schübeler D. MacAlpine D. M. Scalzo D. Wirbelauer C. Kooperberg C. van Leeuwen F. (2004). The histone modification pattern of active genes revealed through genome-wide chromatin analysis of a higher eukaryote. *Gene Dev.* 18 1263–1271. 10.1101/gad.1198204 15175259 PMC420352

[B168] Seto E. Yoshida M. (2014). Erasers of histone acetylation: the histone deacetylase enzymes. *Cold Spring Harb. Perspect. Biol.* 6:a018713. 10.1101/cshperspect.a018713 24691964 PMC3970420

[B169] Shalem O. Sanjana N. E. Zhang F. (2015). High-throughput functional genomics using CRISPR–Cas9. *Nat. Rev. Genet.* 16 299–311. 10.1038/nrg3899 25854182 PMC4503232

[B170] Shen C. Jo S. Y. Liao C. Hess J. L. Nikolovska-Coleska Z. (2013). Targeting recruitment of disruptor of telomeric silencing 1-like (DOT1L): characterizing the interactions between DOT1L and mixed lineage leukemia (MLL) fusion proteins. *J. Biol. Chem.* 288 30585–30596. 10.1074/jbc.m113.457135 23996074 PMC3798529

[B171] Shi J. Vakoc C. R. (2014). The mechanisms behind the therapeutic activity of BET bromodomain inhibition. *Mol. Cell* 54 728–736. 10.1016/j.molcel.2014.05.016 24905006 PMC4236231

[B172] Shi J. Whyte W. A. Zepeda-Mendoza C. J. Milazzo J. P. Shen C. Roe J.-S. (2013). Role of SWI/SNF in acute leukemia maintenance and enhancer-mediated *Myc* regulation. *Gene Dev.* 27 2648–2662. 10.1101/gad.232710.113 24285714 PMC3877755

[B173] Shi Y. Lan F. Matson C. Mulligan P. Whetstine J. R. Cole P. A. (2004). Histone demethylation mediated by the nuclear amine oxidase homolog LSD1. *Cell* 119 941–953. 10.1016/j.cell.2004.12.012 15620353

[B174] Slany R. K. (2009). The molecular biology of mixed lineage leukemia. *Haematologica* 94 984–993. 10.3324/haematol.2008.002436 19535349 PMC2704309

[B175] Slany R. K. (2016). The molecular mechanics of mixed lineage leukemia. *Oncogene* 35 5215–5223. 10.1038/onc.2016.30 26923329

[B176] Sorensen P. Chen C. Smith F. Arthur D. Domer P. Bernstein I. (1994). Molecular rearrangements of the *MLL* gene are present in most cases of infant acute myeloid leukemia and are strongly correlated with monocytic or myelomonocytic phenotypes. *J. Clin. Invest.* 93 429–437. 10.1172/jci116978 8282816 PMC293805

[B177] Stass S. Mirro J. Melvin S. Pui C. Murphy S. Williams D. (1984). Lineage switch in acute leukemia. *Blood* 64 701–706. 6590097

[B178] Stein E. Garcia-Manero G. Rizzieri D. A. Tibes R. Berdeja J. G. Savona M. R. (2018). The DOT1L inhibitor pinometostat reduces H3K79 methylation and has modest clinical activity in adult acute leukemia. *Blood* 131 2661–2669. 10.1182/blood-2017-12-818948 29724899 PMC6265654

[B179] Super H. McCabe N. Thirman M. Larson R. Beau L. M. Pedersen-Bjergaard J. (1993). Rearrangements of the *MLL* gene in therapy-related acute myeloid leukemia in patients previously treated with agents targeting DNA-topoisomerase II. *Blood* 82 3705–3711. 8260707

[B180] Szczepański T. Harrison C. J. van Dongen J. J. (2010). Genetic aberrations in paediatric acute leukaemias and implications for management of patients. *Lancet Oncol.* 11 880–889. 10.1016/s1470-2045(09)70369-9 20435517

[B181] Tamai H. Miyake K. Takatori M. Miyake N. Yamaguchi H. Dan K. (2011). Activated K-Ras protein accelerates human MLL/AF4-induced leukemo-lymphomogenicity in a transgenic mouse model. *Leukemia* 25 888–891. 10.1038/leu.2011.15 21311557

[B182] Tkachuk D. C. Kohler S. Cleary M. L. (1992). Involvement of a homolog of Drosophila trithorax by 11q23 chromosomal translocations in acute leukemias. *Cell* 71 691–700. 10.1016/0092-8674(92)90602-9 1423624

[B183] Tomizawa D. Koh K. Sato T. Kinukawa N. Morimoto A. Isoyama K. (2007). Outcome of risk-based therapy for infant acute lymphoblastic leukemia with or without an *MLL* gene rearrangement, with emphasis on late effects: a final report of two consecutive studies, MLL96 and MLL98, of the Japan Infant Leukemia Study Group. *Leukemia* 21 2258–2263. 10.1038/sj.leu.2404903 17690691

[B184] Tsherniak A. Vazquez F. Montgomery P. G. Weir B. A. Kryukov G. Cowley G. S. (2017). Defining a cancer dependency map. *Cell* 170 564–576.e16. 10.1016/j.cell.2017.06.010 28753430 PMC5667678

[B185] Wan L. Wen H. Li Y. Lyu J. Xi Y. Hoshii T. (2017). ENL links histone acetylation to oncogenic gene expression in acute myeloid leukaemia. *Nature* 543 265–269. 10.1038/nature21687 28241141 PMC5372383

[B186] Wang P. Lin C. Smith E. R. Guo H. Sanderson B. W. Wu M. (2009). Global analysis of H3K4 methylation defines MLL family member targets and points to a role for MLL1-mediated H3K4 methylation in the regulation of transcriptional initiation by RNA polymerase II. *Mol. Cell. Biol.* 29 6074–6085. 10.1128/mcb.00924-09 19703992 PMC2772563

[B187] Wang Q. Wu G. Mi S. He F. Wu J. Dong J. (2011). MLL fusion proteins preferentially regulate a subset of wild-type MLL target genes in the leukemic genome. *Blood* 117 6895–6905. 10.1182/blood-2010-12-324699 21518926 PMC3128481

[B188] Winter G. E. Buckley D. L. Paulk J. Roberts J. M. Souza A. Dhe-Paganon S. (2015). Phthalimide conjugation as a strategy for in vivo target protein degradation. *Science* 348 1376–1381. 10.1126/science.aab1433 25999370 PMC4937790

[B189] Xu J. Li L. Xiong J. denDekker A. Ye A. Karatas H. (2016). MLL1 and MLL1 fusion proteins have distinct functions in regulating leukemic transcription program. *Cell Discov.* 2:16008. 10.1038/celldisc.2016.8 27462455 PMC4869169

[B190] Xu Y. Vakoc C. R. (2017). Targeting cancer cells with BET bromodomain inhibitors. *Cold Spring Harb. Perspect. Med.* 7:a026674. 10.1101/cshperspect.a026674 28213432 PMC5495050

[B191] Yagi H. Deguchi K. Aono A. Tani Y. Kishimoto T. Komori T. (1998). Growth disturbance in fetal liver hematopoiesis of Mll-mutant mice. *Blood* 92 108–117. 9639506

[B192] Yang Z. Yik J. Chen R. He N. Jang M. Ozato K. (2005). Recruitment of P-TEFb for stimulation of transcriptional elongation by the bromodomain protein Brd4. *Mol. Cell.* 19 535–545. 10.1016/j.molcel.2005.06.029 16109377

[B193] Yano T. Nakamura T. Blechman J. Sorio C. Dang C. V. Geiger B. (1997). Nuclear punctate distribution of ALL-1 is conferred by distinct elements at the N terminus of the protein. *Proc. Natl. Acad. Sci. U.S.A.* 94 7286–7291. 10.1073/pnas.94.14.7286 9207083 PMC23813

[B194] Yokoyama A. Cleary M. L. (2008). Menin critically links MLL proteins with LEDGF on cancer-associated target genes. *Cancer Cell* 14 36–46. 10.1016/j.ccr.2008.05.003 18598942 PMC2692591

[B195] Yokoyama A. Lin M. Naresh A. Kitabayashi I. Cleary M. L. (2010). A higher-order complex containing AF4 and ENL family proteins with P-TEFb facilitates oncogenic and physiologic MLL-dependent transcription. *Cancer Cell* 17 198–212. 10.1016/j.ccr.2009.12.040 20153263 PMC2824033

[B196] Yokoyama A. Somervaille T. Smith K. S. Rozenblatt-Rosen O. Meyerson M. Cleary M. L. (2005). The menin tumor suppressor protein is an essential oncogenic cofactor for MLL-associated leukemogenesis. *Cell* 123 207–218. 10.1016/j.cell.2005.09.025 16239140

[B197] Yu B. D. Hess J. L. Horning S. E. Brown G. A. Korsmeyer S. J. (1995). Altered *Hox* expression and segmental identity in *Mll*-mutant mice. *Nature* 378 505–508. 10.1038/378505a0 7477409

[B198] Zeisig D. T. Bittner C. B. Zeisig B. B. García-Cuéllar M.-P. Hess J. L. Slany R. K. (2005). The eleven-nineteen-leukemia protein ENL connects nuclear MLL fusion partners with chromatin. *Oncogene* 24 5525–5532. 10.1038/sj.onc.1208699 15856011

[B199] Zeleznik-Le N. Harden A. Rowley J. (1994). 11q23 translocations split the “AT-hook” cruciform DNA-binding region and the transcriptional repression domain from the activation domain of the mixed-lineage leukemia (*MLL*) gene. *Proc. Natl. Acad. Sci. U.S.A.* 91 10610–10614. 10.1073/pnas.91.22.10610 7938000 PMC45071

[B200] Zhang T. Cooper S. Brockdorff N. (2015). The interplay of histone modifications – writers that read. *EMBO Rep.* 16 1467–1481. 10.15252/embr.201540945 26474904 PMC4641500

[B201] Zhao Z. Wang L. Volk A. G. Birch N. W. Stoltz K. L. Bartom E. T. (2018). Regulation of MLL/COMPASS stability through its proteolytic cleavage by taspase1 as a possible approach for clinical therapy of leukemia. *Gene Dev.* 33 61–74. 10.1101/gad.319830.118 30573454 PMC6317322

[B202] Zhu L. Li Q. Wong S. Huang M. Klein B. J. Shen J. (2016). ASH1L links histone H3 lysine 36 dimethylation to MLL Leukemia. *Cancer Discov.* 6 770–783. 10.1158/2159-8290.cd-16-0058 27154821 PMC4930721

[B203] Ziemin-van der Poel S. McCabe N. Gill H. Espinosa R. Patel Y. Harden A. (1991). Identification of a gene, *MLL*, that spans the breakpoint in 11q23 translocations associated with human leukemias. *Proc. Natl. Acad. Sci. U.S.A.* 88 10735–10739. 10.1073/pnas.88.23.10735 1720549 PMC53005

[B204] Zuber J. Shi J. Wang E. Rappaport A. R. Herrmann H. Sison E. A. (2011). RNAi screen identifies Brd4 as a therapeutic target in acute myeloid leukaemia. *Nature* 478 524–528. 10.1038/nature10334 21814200 PMC3328300

